# Robust Identification of Suitable T-Cell Subsets for Personalized CMV-Specific T-Cell Immunotherapy Using CD45RA and CD62L Microbeads

**DOI:** 10.3390/ijms20061415

**Published:** 2019-03-20

**Authors:** Caroline Mangare, Sabine Tischer-Zimmermann, Sebastian B. Riese, Anna C. Dragon, Immo Prinz, Rainer Blasczyk, Britta Maecker-Kolhoff, Britta Eiz-Vesper

**Affiliations:** 1 Institute for Transfusion Medicine, Hannover Medical School, 30625 Hannover, Germany; mangare.caroline@mh-hannover.de (C.M.); tischer-zimmermann.sabine@mh-hannover.de (S.T.-Z.); riese.sebastian@mh-hannover.de (S.B.R.); dragon.anna@mh-hannover.de (A.C.D.); blasczyk.rainer@mh-hannover.de (R.B.); 2 Integrated Research and Treatment Center (IFB-Tx), Hannover Medical School, 30625 Hannover, Germany; maecker-kolhoff.britta@mh-hannover.de; 3 Institute of Immunology, Hannover Medical School, 30625 Hannover, Germany; prinz.immo@mh-hannover.de; 4 Department of Pediatric Hematology and Oncology, Hannover Medical School, 30625 Hannover, Germany

**Keywords:** cytomegalovirus (CMV), donor lymphocyte infusions (DLIs), graft versus host disease (GvHD), naive T-cell depletion

## Abstract

Viral infections and reactivations remain a serious obstacle to successful hematopoietic stem cell transplantation (HSCT). When antiviral drug treatment fails, adoptive virus-specific T-cell transfer provides an effective alternative. Assuming that naive T cells (T_N_) are mainly responsible for GvHD, methods were developed to generate naive T-cell-depleted products while preserving immune memory against viral infections. We compared two major strategies to deplete potentially alloreactive T cells: CD45RA and CD62L depletion and analyzed phenotype and functionality of the resulting CD45RA^−^/CD62L^−^ naive T-cell-depleted as well as CD45RA^+^/CD62L^+^ naive T-cell-enriched fractions in the CMV pp65 and IE1 antigen model. CD45RA depletion resulted in loss of terminally differentiated effector memory T cells re-expressing CD45RA (T_EMRA_), and CD62L depletion in loss of central memory T cells (T_CM_). Based on these differences in target cell-dependent and target cell-independent assays, antigen-specific T-cell responses in CD62L-depleted fraction were consistently 3–5 fold higher than those in CD45RA-depleted fraction. Interestingly, we also observed high donor variability in the CD45RA-depleted fraction, resulting in a substantial loss of immune memory. Accordingly, we identified donors with expected response (DER) and unexpected response (DUR). Taken together, our results showed that a naive T-cell depletion method should be chosen individually, based on the immunophenotypic composition of the T-cell populations present.

## 1. Introduction

Hematopoietic stem cell transplantation (HSCT) is a curative therapeutic approach for several hematologic and non-hematologic disorders. Infectious complications due to delayed immune reconstitution or pharmacological immunosuppression increase the morbidity and mortality of transplant patients. Recipients are highly susceptible to common viral infections and reactivations by lytic pathogens like human adenovirus, endogenous herpes viruses like Epstein–Barr virus (EBV), cytomegalovirus (CMV), human herpesvirus 6 (HHV6), and BK polyomavirus [1]. Initial reduction of immunosuppression has become a standard approach to restoring antiviral immunity. Antiviral drugs such as foscarnet, valganciclovir, ganciclovir, letermovir [2], or brincidofovir [3] are administered to prevent and control viral infection and reactivation [4,5]. Unfortunately, these drugs are associated with downstream complications such as significant toxicity. Furthermore, the evolution of isolates resistant to letermovir has been described. This recently approved anti-CMV agent acts by inhibiting CMV replication by binding to terminase complex proteins pUL51 and/or pUL56 as well as by cleaving concatemeric genomic viral DNA [4,6,7].

Consequently, many groups have explored the therapeutic potential of virus-specific memory T cells (VSTs) [8,9], which can be transferred through unmanipulated donor lymphocyte infusions (DLIs) from seropositive donors. Nevertheless, DLIs were found to be associated with risk of graft versus host disease (GvHD), which further affects immune recovery due to the need to increase immunosuppression [10,11]. Therefore, different strategies to specifically isolate VSTs from seropositive donors for clinical use have been developed during the last two decades. Current strategies include: (1) enrichment via an interferon-γ (IFN-γ) cytokine capture system (CCS) [12,13,14]; (2) reversible peptide-MHC (pMHC) multimers [15,16,17]; as well as (3) in vitro expansion from a small number of precursor cells in the presence of specific antigens and different cytokine combinations [18,19]. All of the above-mentioned strategies have achieved promising viral eradication results [20,21,22,23]. Nonetheless, the ability to generate these antiviral memory T-cell products is limited as they require knowledge of immunodominant viral epitopes and the availability of good manufacturing practice (GMP)-quality grade antigens for stimulation and enrichment [20]. Other problems include the high antigenic diversity of viruses due to different protein expression and human leukocyte antigen (HLA)-restrictions and the low immunogenicity of some T-cell epitopes, e.g., Epstein-Barr nuclear antigen 1 (EBNA1) [24].

Naive T-cell (T_N_) depletion procedures were established to overcome these limitations and to provide a broad spectrum of antiviral T cells for patients suffering from multiple viral complications and infections for which no GMP-grade immunodominant antigens are available (e.g., HHV6). These T-cell products preserve memory T-cell responses against viral infections [25,26] by concurrently suppressing the incidence of GvHD [27,28,29,30,31]. The presence of naive T cells in T-cell products precipitates the risk of GvHD. Naive T cells have a typically broader T-cell receptor (TCR) repertoire and, therefore, a higher alloreactivity potential than memory T-cell fractions [32,33]. Naive CD4^+^ and CD8^+^ T cells are characterized by their surface expression of CD45RA, adhesion molecules CD62L and C-C chemokine receptor 7 (CCR7), allowing for cell depletion using antibodies against these markers. Methods for immunomagnetic depletion of T_N_ using CD45RA microbeads [26,27,28,29,30] and CD62L microbeads [31], respectively, have been developed and shown to be effective. 

The T-cell phenotype is characterized as follows: naive T cells (T_N_) (CD45RA^+^CD45RO^−^CCR7^+^CD62L^+^CD27^+^CD28^+^), central memory T cells (T_CM_) (CD45RA^−^CD45RO^+^CCR7^+^CD62L^+^CD27^+^CD28^+^), effector memory T cells (T_EM_) (CD45RA^−^CD45RO^+^CCR7^−^CD62L^−^CD27^+^CD28^−^), and late effector memory T cells re-expressing CD45RA (T_EMRA_) (CD45RA^+^CD45RO^−^CCR7^−^CD62L^−^CD27^−^CD28^−^) [34]. In-depth analysis in various studies has shown a strong correlation between the phenotypic profile of antiviral T cells and their protective efficacy against different viruses in vivo [35]. Chronic viral infections exhibit great phenotypic T-cell heterogeneity and T-cell phenotypes predominantly found during latent infection are: hepatitis C virus (HCV) (CCR7^+^CD27^+^CD28^+^), EBV (CCR7^−^CD27^+^CD28^+^), human immunodeficiency virus (HIV) (CD45RA^−^CCR7^−^CD27^+^CD28^+^) [36], and CMV (CD45RA^+^CCR7^−^CD27^−^CD28^−^) [35,37,38]. Beyond T-cell phenotype, associations between the functional attributes of CD4^+^ or CD8^+^ antiviral T cells among different pathogens have been reported [34,39]. T-cell responses against CMV and EBV are mainly controlled by cytotoxic CD8^+^ T cells, whereas the presence of CD4^+^ T helper cells is essential for the defense against adenovirus [40] and Dengue virus [41]. 

In view of this heterogeneity, we hypothesize that T-cell depletion strategies covering a single cell surface epitope such as CD45RA or CD62L might also result in the depletion of antiviral effector T-cell populations [31]. To prove this hypothesis, we designed the present study to test for associations between T_N_ depletion using CD45RA and CD62L microbeads and the resulting CMV-specific T-cell response dynamics. CMV is a ubiquitous virus with a wide range of clinical presentations. Infection with CMV is often asymptomatic in healthy individuals [42]. CMV infection or reactivation is the most common opportunistic infection in immunocompromised patients, and is still associated with increased transplant-related mortality [43,44,45]. Since CMV-specific T-cell response embodies a heterogeneous mixture of distinct and mainly CD8^+^ T-cell phenotypes with different functions [37,46], we aimed to comprehensively investigate the effect of T_N_ depletion on the phenotype and functionality of the resulting CMV-specific memory T-cell fraction as well as the inferred naive T-cell fraction. To identify potential correlations of CMV response among naive and memory T-cell fractions, we evaluated T-cell phenotypes, frequencies, and functional properties in vitro using ELISpot and intracellular cytokine staining (ICS) assays. 

Our results showed that the presence of effector memory T cells, particularly T_EMRA_, in the CD62L-negative fraction (CD62L_NF) and that depletion of the latter in the CD45RA-negative fraction (CD45RA_NF) contributed significantly to the overall CMV-specific T-cell response against the immunodominant pp65 and immediate-early protein 1 (IE1) antigens. The CMV-specific T_EMRA_ population present in the naive CD45RA_PF fraction is highly cytotoxic and was not present in the CD45RA_NF. Furthermore, we found strong inter-individual differences in T-cell responses, which we used to classify donors with expected response (DER) from donors with unexpected response (DUR). We were able to explain the observed T-cell responses based on this classification and by analyzing the T-cell phenotypes in theses donor cohorts separately. These findings show that CMV-specific T-cell responses are, in large part, determined by the specificity and phenotype of the corresponding immune responses in the donor. Our data underline the need to select precise methods for T_N_ depletion in order to provide effective antiviral memory T cells for clinical applications.

## 2. Results

### 2.1. Cell Selection Strategy for Naive T-Cell Depletion Using CD45RA and CD62L Microbeads

In order to select the most suitable columns for the depletion of naive T cells, we first compared the efficacy of LS^+^ and LD^−^ columns using CD45RA and CD62L microbeads, respectively. Peripheral blood mononuclear cells (PBMCs) and CD3^+^ T cells were used as starting populations. The fractions obtained were termed and gated as follows: memory CD3^+^CD45RA^−^ (CD45RA_NF) or memory CD3^+^CD62L^−^ T cells (CD62L_NF) and naive CD3^+^CD45RA^+^ (CD45RA_PF) or naive CD3^+^CD62L^+^ (CD62L_PF) (Supplementary Figure S1A–E).

PBMCs consisted of CD3^+^ T cells (mean of 43.30%, range 40.30–47.80%) of the following composition: 26.93% (23.7–32.0%) were CD45RA^+^ T cells and 73.1% (68.0–76.4%) were memory CD45RA^−^ T cells whereas 78.17% (71.10–82.40%) were CD62L^+^ T cells and 21.87% (17.60–28.90%) were memory CD62L^−^ T cells. The mean phenotypic composition on CD3^+^ T cells within the PBMCs is shown in Supplementary Figure S2A,C and Supplementary Table S1A. Regarding CD45RA depletion using LS^+^ versus LD^−^ columns, there were no significant differences in the overall purity between CD3^+^CD45RA_NF fractions obtained with LS^+^ columns (28.13%) and LD^−^ columns (23.87%). The phenotypic composition of CD3^+^ T cells in the CD45RA_NF after LS^+^ column-based depletion consisted mainly of T_CM_ and T_EM_ and did not differ significantly between the columns. Differences were recorded among CD45RA_PF obtained using the LS^+^ columns which composed mainly of T_N_ and T_EMRA_ (Supplementary Figure S2A). Following depletion on LD^−^ columns, however, substantial amounts of all four T-cell subsets were found_._ Furthermore, the absolute cell numbers obtained in CD45RA_NF using LS^+^ column had approximately 7.2-fold loss in cell recovery of expected cell amount and even lower approximately 4.6-fold loss using LD^−^ columns (Supplementary Figure S2B). 

Following CD62L depletion using PBMCs from the same donors there were no significant differences in the overall purity between CD3^+^ T cells obtained with LS^+^ columns versus LD^−^ columns; the mean frequencies were 29.0% and 25.80%, respectively (Supplementary Table S2A). The resulting phenotypic composition of the CD62L_NF for LS^+^ and LD^−^ columns comprised mainly of T_EM_ and T_EMRA_ (Supplementary Figure S2C, Supplementary Table S1A). Surprisingly, following depletion with LS^+^ and LD^−^ columns, there were no notable differences in the composition of CD62L_PF which was composed mainly of T_N_ and T_CM_. The cell recovery from LS^+^ and LD^−^ columns were similar (Supplementary Figure S2D, Supplementary Table S1A).

In order to increase the yield and purity of memory T cells for functional assays, isolated CD3^+^ T cells were used as starting material, and depletion of CD45RA^+^ naive T cells was performed as a proof of concept (Supplementary Figure S2E–F). The CD3^+^ T-cell fraction had a mean purity of 97.05% (96.25–98.3%); 41.23% (35.7–42.2%) of the T cells were CD45RA^+^ and 58.77% (54.1–64.4%) were CD45RA^−^ (Supplementary Table S2B). As expected, the overall purity of CD3^+^ T cells in the CD45RA_NF obtained using LS^+^ and LD^−^ columns was high: a mean of 98.77% (98.6–99.0%) and 96.67% (93.4–98.5%), respectively. Regarding phenotypic composition, the CD45RA_NF obtained with LS^+^ columns was comparable with LD^−^ columns and comprised mainly of T_CM_ and T_EM_. The CD45RA_PF obtained by LS^+^ column-based depletion was composed mainly of T_N_ and T_EMRA,_ on the other hand, depletion with LD^−^ columns resulted in substantial frequencies of all four T-cell phenotypes (Supplementary Figure S2E).

Although the T-cell purities of CD45RA_NF following depletion with LS^+^ and LD^−^ columns were comparable, strong differences in the phenotypic composition of the naive CD45RA_PF fractions were observed, which were further reflected by drastic differences in the amount of cell recovery (Supplementary Figure S2F). The use of LS^+^ columns resulted in cell recovery amounting to a 1.7-fold loss of the expected amount and a mean yield of 59.9%. The usage of LD^−^ columns resulted in cell recovery of approximately 2.5-fold loss of the expected amount of cells, with a mean yield of 20.3%. Based on these results, notably with respect to the recovery of memory T cells in the CD45RA_NF, further experiments were performed using isolated CD3^+^ T cells and LS^+^ columns.

### 2.2. Multiparametric Evaluation of Cell Fractions Following Naive T-Cell Depletion with CD45RA and CD62L Microbeads

**Figure 1:**
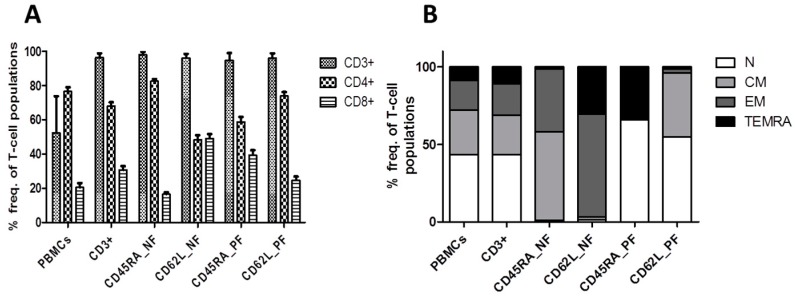
Evaluation of T-cell frequencies and phenotypes following naive T-cell depletion. CD3+ T cells were isolated from PBMCs from 24 healthy donors and naive T-cell depletion was performed using CD45RA and CD62L microBeads (Miltenyi Biotec). Immunophenotypic analysis was performed by flow cytometry before and after depletion. (**A**) Frequencies of CD3^+^, CD4^+^, and CD8^+^ T cells within the different T-cell fractions; (**B**) T-cell phenotypes, as determined by gating CD45RA against CD62L and dividing cells into the following subsets: naive (T_N_: CD45RA^+^CD62L^+^), central memory (T_CM_: CD45RA^−^CD62L^+^), effector memory (T_EM_: CD45RA^−^CD62L^−^), and late effector memory T cells re-expressing CD45RA (T_EMRA_: CD45RA^+^CD62L^−^). The isolated T-cell fractions consisted mainly of a CD45RA_PF (T_N_ and T_EMRA_) and a CD45RA_NF fraction (T_CM_ and T_EM_) as well as a CD62L_PF (T_N_ and T_CM_) and a CD62L_NF fraction (T_EMRA_ and T_EM_). Data represent the means of 24 donors. NF: negative fraction (memory); PF: positive fraction (naive)

The mean T-cell frequencies and phenotypes within all the T-cell fractions in samples from 24 donors are shown in Table 1 and Figure 1.

Mean T-cell frequencies in the CD45RA_NF and CD62L_NF memory fractions were 98.08% and 96.19% in the CD3^+^ T-cell subsets, 82.81% and 48.98% in the CD4^+^ T-cell subsets and 16.34% and 48.47% in the CD8^+^ T-cell subsets, respectively (Table 1, Figure 1A). Hence, the depletion and loss of CD8^+^ T cells was clearly greater in the CD45RA_NF than in the CD62L_NF. Subsequent analysis showed that the CD4/CD8 ratio increased to 5.08 in the CD45RA_NF and decreased to 1.01 in the CD62L_NF (Table 1). The corresponding CD45RA_PF and CD62L_PF naive fractions had mean T-cell frequencies of 94.83% and 96.11% in the CD3^+^ T-cell subsets, 59.84% and 73.99% in the CD4^+^ T-cell subsets, and 38.15% and 24.66% in the CD8^+^ T cells, respectively. Based on the preceding results (Supplementary Figure S2), we expected to see differences in CD3^+^ T-cell phenotypes within the CD45RA_NF and CD62L_NF memory fractions (Table 1, Figure 1B). The memory CD45RA_NF was comprised mainly of T_CM_ and T_EM_, whereas the memory CD62L_NF consisted of mainly T_EM_ and T_EMRA_. Conversely, the naive CD45RA_PF contained predominantly T_N_ and T_EMRA_, while the naive CD62L_PF contained T_N_ and T_CM_. Overall, the analysis of T-cell frequencies and phenotypes showed that manual naive T-cell depletion using CD45RA and CD62L microbeads is feasible and yields memory T-cell subsets of high purity. Hence, the predominant T-cell phenotypes were T_CM_ and T_EM_ in the memory CD45RA_NF, T_EM_ and T_EMRA_ in the memory CD62L_NF, T_N_ and T_EMRA_ in the naive CD45RA_PF and T_N_ and T_CM_ in the naive CD62L_PF.

### 2.3. T-Cell Response to ppCMV_pp65 by IFN-γ Enzyme-Linked ImmunoSpot (ELISpot) Assay

**Figure 2:**
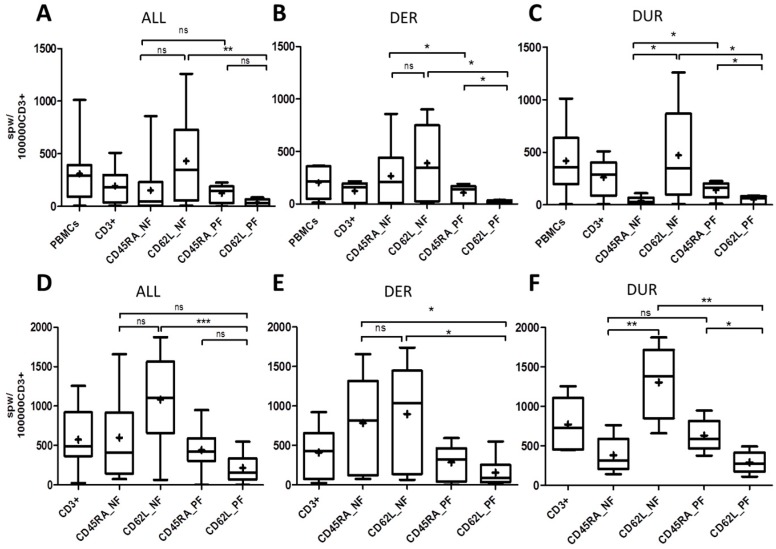
T-cell response to ppCMV_pp65 using ELISpot assay. CMV-specific T cells were determined by IFN-γ ELISpot in a target cell-independent assay (**A–C**) and a target cell-dependent assay (**D–F**). (**A–C**) CMV-specific T-cell frequencies detected by IFN-γ secretion after stimulating the PBMCs (2.5 × 10^5^ cells) and the different T-cell fractions (5 × 10^4^ effector cells) with 1 μg/µL ppCMV_pp65 (target cell-independent assay). (**D–F**) The target cell-dependent assay utilized loaded target cells, which consisted of CD3-negative cells (target cells) stimulated overnight with 1 μg/µL ppCMV_pp65 (1 × 10^7^ cells). The loaded ELISpot plates were incubated for 16 h, spots were developed and analyzed according to the manufacturer’s instructions. (**A**) and (**D**) show results for all donors (ALL), (**B**) and (**E**) for donors with expected response (DER), and (**C**) and (**F**) for donors with unexpected response (DUR). Data are expressed as means of 12 donors and were calculated by subtracting the observed values from the negative controls. The number of spots per well (spw) was normalized to 100,000 CD3^+^ T cells (spwT). Responder groups were classified based on CMV-specific T-cell responses obtained within the CD45RA_NF/CD45RA_PF memory and naive fractions, respectively and were determined per individual donor. Whiskers and boxes show maximum and minimum values and a plus sign represents the mean. Asterisks indicate statistically significant differences (A and D: Kruskal–Wallis test, B, C, E, and F: unpaired Student’s *t*-test, * *p* < 0.05, ** *p* < 0.01, *** *p* < 0.001, not significant (ns)).

T-cell responses of the resulting CD45RA_PF/NF and CD62L_PF/NF were evaluated in 12 different CMV-seropositive donors. The mean T-cell frequencies and phenotypes detected in these donors are shown in Table 2. CMV-specific T-cell responses were assessed by target cell-independent and target cell-dependent IFN-γ ELISpot assay upon in vitro stimulation with overlapping peptide pools of ppCMV_pp65 and ppCMV_IE1. Overall, the target cell-independent IFN-γ ELISpot assay identified higher ppCMVpp65 T-cell responses in the memory fractions CD45RA_NF with a mean of 151.4 spots per well (spw)/100,000 CD3^+^ T cells (spwT), and 430.4 spwT in CD62L_NF than in the naive fractions CD45RA_PF with 123.8 spwT and the least in CD62L_PF with 34.88 spwT (Figure 2A).

Paradoxically, in depth analysis revealed that only 6/12 donors (50%) had higher ppCMV_pp65 T-cell responses in the memory CD45RA_NF had 265.04 spwT than in the corresponding naive CD45RA_PF had 107.02 spwT (Figure 2B). Accordingly, these donors were classified as “donors with expected response” (DER). On the contrary in the remaining 6/12 (50%) donors, the response in the inferred naive CD45RA_PF had 140.56 spwT was higher than that in the corresponding memory CD45RA_NF had 37.71 spwT (Figure 2C). These donors were thus referred to as “donors with unexpected response” (DUR). Interestingly, these differences in T-cell responses were only observed in the CD45RA fraction. 

Further analysis showed that both DER and DUR donors always had considerably higher responses in the memory CD62L_NF than the corresponding CD62L_PF (Figure 2B,C). These target cell-independent results were confirmed by the results of a target cell-dependent assay with an effector-target (E:T) ratio of 1:1 (Figure 2D–F). In general, a similar pattern of response was observed among all T-cell fractions. The highest CMV-specific T-cell responses were observed in the memory fractions: the CD45RA_NF and those in the CD62L_NF were even higher (Figure 2D). The lowest CMV-specific T-cell responses were observed in the naive fractions. 

In-depth analyses to confirm the previously determined DER and DUR donor classifications were performed in a similar manner. Similarly, DER had higher responses in both memory fractions (Figure 2E) than in the naive fractions. DUR, on the other hand, had higher T-cell responses in the naive CD45RA_PF than in the memory CD45RA_NF (Figure 2F) and higher responses in the memory CD62L_NF than in the naive CD62L_PF. 

**Figure 3:**
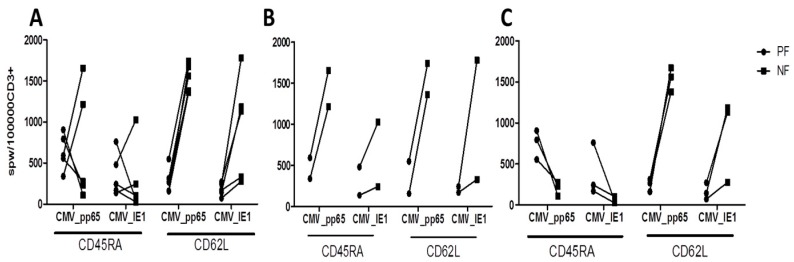
ppCMV_pp65 induced more IFN-γ secretion than ppCMV_IE1, as shown by target cell-dependent IFN-γ ELISpot assay. The assay utilized loaded target cells consisting of CD3 negative cells stimulated overnight with 1 μg/µL ppCMV_pp65 or ppCMV_IE1 incubated at an effector: target ratio of 1:1 for 16 h. Results are shown for (**A**) all donors (ALL), (**B**) donors with expected response (DER), and (**C**) donors with unexpected response (DUR). The T-cell fractions of each donor showed similar patterns against target cells loaded with ppCMV_pp65 and ppCMV_IE1. NF: negative fraction (memory); PF: positive fraction (naive)

Additionally, a target cell-dependent assay was performed with an E:T ratio of 2:1 and the results were compared with those of the assay with an E:T ratio of 1:1 (Supplementary Figure S3). Again, T-cell responses were comparably higher in the memory fractions of CD45RA_NF and CD62L_NF (Supplementary Figure S3A) and lower in the naive fractions. Similarly, DER showed higher responses in the memory CD45RA_NF than in the naive CD45RA_PF (Supplementary Figure S3B), whereas DUR exhibited higher T-cell responses in the naive CD45RA_PF than in the memory CD45RA_NF (Supplementary Figure S3C). As before, CMV T-cell responses were always higher in the memory CD62L_NF with a mean of 548.42 spwT in DER and 572.73 spwT in DUR than in the naive CD62L_PF with 46.72 spwT in DER and 268.62 spwT in DUR. Additional target-cell dependent experiments were performed in parallel following stimulation of 5/12 donors with ppCMV_IE1 and ppCMV_pp65, respectively (Figure 3).

Stimulation with ppCMV_pp65 and ppCMV_IE1 resulted in similar patterns of T-cell responses. These donors showed higher T-cell response against ppCMV_pp65 than to ppCMV_IE1. Overall, the results for the CD45RA_NF and CD45RA_PF were comparable 350.80 spwT versus 375.0 spwT. The highest ppCMV_IE1 responses were found in the memory CD62L_NF with 982.8 spwT while the lowest in the naive CD62L_PF 123.9 spwT. The donors classified according to response against ppCMV_pp65 (Figure 2) maintained this responder status in response to ppCMV_IE1 (Figure 3). In DER, CMV_IE1-specific T-cell responses were higher in the memory fractions (Figure 3B) than in the naive fractions. In DUR, on the other hand, the responses were higher in the naive CD45RA_PF than in the memory CD45RA_NF (Figure 3C), and much higher in the memory CD62L_NF than in the naive CD62L_PF.

In summary, since the responses in the memory CD45RA_NF were not as high as expected compared to those in the naive CD45RA_PF, we classified the donors into two categories: donors with expected response (DER) and donors with unexpected response (DUR). Only DUR showed significant differences between the memory CD45RA_NF and the naive CD45RA_PF in the target cell-independent assay and the target cell-dependent assay, in which T-cell responses were higher in the CD45RA_PF. Interestingly, CMV-specific T-cell responses (spwT) were always robust and consistently higher in the memory CD62L_NF than in the naive CD62L_PF in both donor categories and in both the target cell-independent and the target cell-dependent assay (Figure 2 and Figure 3). Additionally, the CD62L_NF memory fraction exhibited significantly higher anti-CMV T-cell reactivity than the CD45RA_NF memory fraction in all donors in both assays (target cell-independent and -dependent). Furthermore, the CD62L_PF naive fraction had lower CMV-specific T-cell response rates than the CD45RA_PF naive fraction in both donor categories and assays (target cell-independent and -dependent). The differences in CMV-specific T-cell responses between the CD45RA_NF/CD45RA_PF among the donors could reflect intrinsic donor factors; we performed further analyses to investigate this issue.

### 2.4. Correlations between T-Cell Frequencies, Phenotypes and Auxiliary T Cells among Donors with Expected and Unexpected Response

**Figure 4:**
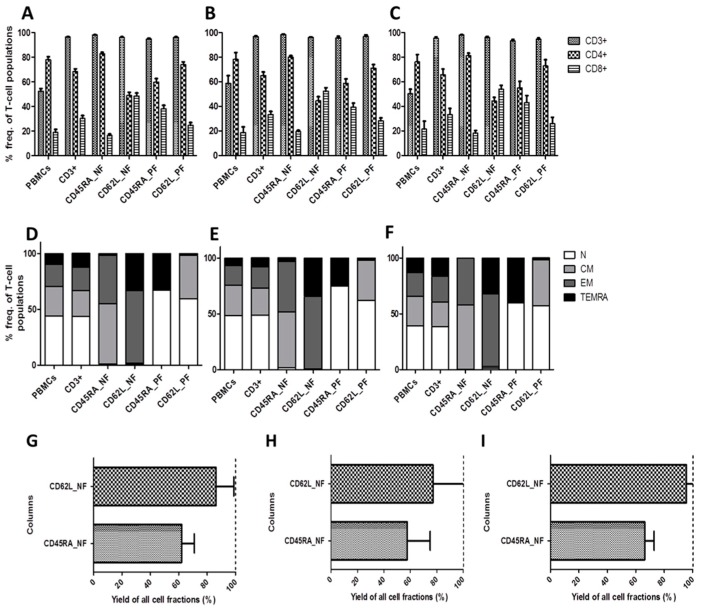
Evaluation of T-cell frequencies, phenotypes and yields among expected and unexpected responders. Mean and standard deviation of T-cell counts of T-cell fractions for (**A**) all donors (ALL); (**B**) donors with expected response (DER); and (**C**) donors with unexpected response (DUR) are shown. Mean T-cell phenotype gated among CD3^+^ T-cell populations for (**D**) all donors, (**E**) DER, and (**F**) DUR. Yields of CD62L^−^ and CD45RA^−^ T cells in the CD62L_NF and CD45RA_NF memory fractions were calculated to 100% purity from the starting fractions and shown for (**G**) all donors, (**H**) DER, and (**I**) DUR. Data represent the mean ± SD of *n* = 12 donors. The dotted line stands for the expected yield.

Due to the observed differences in CMV-specific T-cell responses between donors and their consequent classification as donors with expected or unexpected response (DER and DUR), we comprehensively analyzed and dissected the phenotypic compositions and T-cell frequencies of each subgroup (Table 2, Figure 4, Supplementary Figure S4, Supplementary Table S2A,B).

Overall, the CD45RA_NF and CD62L_NF memory fractions were dominated by T_CM_ and T_EM_ (CD45RA_NF) and T_EM_ and T_EMRA_ (CD62L_NF). In detail, the mean T-cell frequencies of the predominant T-cell populations within the memory fractions of CD45RA_NF and CD62L_NF as well as naive CD45RA_PF and CD62L_PF among CD3^+^, CD4^+^ and CD8^+^ subset are as shown in Table 2, Figure 4A,D, Supplementary Figure S4A,E, Supplementary Table S2A,B. 

In DER, the memory CD45RA_NF contained predominantly T_CM_ 50.23% and T_EM_ 45.15% within the CD3^+^ T-cell subset, while the naive CD45RA_PF contained mainly T_N_ 74.92% and T_EMRA_ 23.68%. In DUR, on the other hand, the memory CD45RA_NF contained mainly T_CM_ 57.93% and T_EM_ 41.82%, while the naive CD45RA_PF contained T_N_ 59.65% and T_EMRA_ 39.05%. Generally speaking, DUR samples contained slightly more memory T cells (99.75%) than DER (95.38%). We also performed separate in-depth analyses of the CD8^+^ and CD4^+^ T-cell subsets and the two donor categories (DER and DUR). CD8^+^ T-cell subset analysis revealed that the memory CD45RA_NF in DER contained 28.7% T_CM_ and 70.08% T_EM_ CD8^+^ T cells (total memory cells: 98.78%) compared to 34.4% T_CM_ and 64.05% T_EM_ in CD8^+^ T cells (total of memory cells: 99.5%) in DUR. As the total number of memory T cells is almost equal, the higher T-cell response in CD45RA_NF suggests that the observed differences could be due to high amount of CD8^+^ T_EM_ 70.08% in DER and 65.07% in DUR. While the higher T-cell response in CD45RA_PF could be due to high amount of CD8^+^ T_EMRA_ 66.93% in DUR and 44.95% in DER. In the CD62L_NF memory fraction, on the other hand, DER had higher frequencies of T_EMRA_ 52.37% than DUR 46.22% in the CD8^+^ T-cell subset. (Supplementary Figure S4, Supplementary Table S2A,B). The memory CD62L_NF was consistently related to higher CMV-specific T-cell responses than the naive CD62L_PF. 

Due to the role of naive T cells in causing GvHD, we evaluated the residual T_N_ frequencies within the CD8^+^ and CD4^+^ T-cell subsets of the memory fractions to determine where they predominate. The memory CD45RA_NF contained similar numbers of naive CD8^+^ T cells with 0.44% and CD4^+^ T cells with 0.33%. Similar frequencies were observed in DER and DUR samples. The memory CD62L_NF exhibited more naive T cells within the CD4^+^ T-cell subset: 2.07% than within the CD8^+^ T-cell subset: 0.92% in both DER and DUR combined (Supplementary Figure S4, Table 2). Nevertheless, naive fractions also contained memory T cells due to co-expression of the depletion markers on varying populations of memory T cells, as shown in Table 2, Supplementary Figure S4 and Supplementary Table S2A,B. For instance, within the naive CD45RA_PF, the majority of T_EMRA_ were found within the CD8^+^ T-cell subset: 55.94%, and only 14.03% within the CD4^+^ T-cell subset. Similarly, CD8^+^ T_EMRA_ frequencies as high as 44.95% and 66.93% compared to CD4^+^ T_EMRA_ frequencies of 11.97% and 17.67% were observed in DER and DUR, respectively. Overall, mean T_EMRA_ frequencies were higher in DUR (84.6%) than in DER (58.98%). Therefore, the higher frequencies of T_EMRA_ observed in the naive CD45RA_PF of DUR could have contributed to the unexpected finding of higher CMV-specific T-cell responses in this donor category. It is also noteworthy that phenotypic analysis of CD4^+^ and CD8^+^ T cells revealed that a higher population of CD8^+^ T_EMRA_ correlated with a decrease in the CD8^+^ T_CM_ population and with an increase in the CD4^+^ T_CM_ population (Supplementary Figure S4). 

Furthermore, we observed that CD45RA depletion led to increased enrichment of CD4^+^ T cells in conjunction with substantial depletion of CD8^+^ T cells in the memory T-cell compartment (Table 2, Supplementary Table S2A,B). In the memory CD62L_NF, on the other hand, there was a slight increase of CD8^+^ T cells (which might explain why we observed high CMV-specific T-cell responses), whereas the naive CD62L_PF had greater enrichment of CD4^+^ T cells and the lowest CMV-specific T-cell responses. Interestingly, the naive CD62L_PF showed the lowest CMV-specific T-cell responses despite high frequencies of T_CM_ 45.72% for CD4^+^ T_CM_ and 27.78% for CD8^+^ T_CM_. Similarities between T_CM_ distribution frequencies were observed in the memory CD45RA_NF and the naive CD62L_PF where these cells occurred predominantly within the CD4^+^ T-cell subsets, irrespective of the donor response classification. The fact that CD4^+^ T cells are not more cytotoxic than CD8^+^ T cells could partly explain why the observed responses in these categories were not as robust as anticipated, despite the high frequencies of T_CM_. These results indicate that deeper phenotypic analysis can be used to predict the T-cell response outcomes.

**Figure 5:**
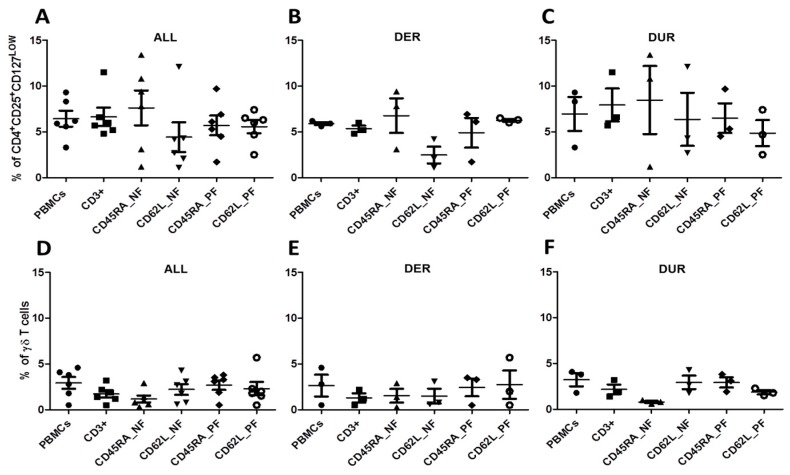
Frequency of auxiliary Tregs and γδ T cells among different T-cell fractions Flow cytometry was used to determine the frequency of Tregs (CD4^+^CD25^+^CD127^low^) and TCR γδ T cells for (**A**) all donors (ALL), (**B**) donors with expected response (DER), and (**C**) donors with unexpected response (DUR). Frequencies of γδ T cells gated among CD3^+^ T cells for (**D**) all donors, (**E**) DER and (**F**) DUR. Data represent the mean ± SD of *n* = 12 donors.

Due to their potential to influence antiviral CMV_pp65-specific T-cell responses and to suppress GvHD, we also evaluated the frequencies of auxiliary γδ T cells and Tregs (CD4^+^CD25^+^CD127^low^) in samples from eight patients (Table 2, Figure 5).

Relative to the starting fraction of CD3^+^ T cells 6.63%, the frequency of Tregs among the memory CD45RA_NF was slightly higher 7.62%. Interestingly, the frequency of Tregs in the memory CD62L_NF was the lowest 4.42%, and this population generally exhibited the strongest T-cell response (Table 2, Figure 2A,5A). The CD45RA_PF and CD62L_PF naive fractions had similar Treg frequencies: 5.7% and 5.57% respectively. The mean frequencies of Tregs within DER and DUR are shown in Supplementary Table S2A,B, Figure 5B,C. Our results for Treg frequencies did not explain the differences in responses between donors. 

Compared to the overall frequency of 1.73% CD3^+^ γδ T cells in the starting population, the number in the memory CD45RA_NF slightly decreased to 1.16% while increases were observed in the CD62L_NF memory fraction 2.22% as well as in the CD45RA_PF 2.68% and CD62L_PF naive fractions 2.30% (Table 2, Figure 5D). The mean frequencies of γδ T cells within DER and DUR are shown in Supplementary Table S2A,B, Figure 5E,F. In DUR, on the other hand, the highest γδ T-cell frequencies were found in the CD62L_NF memory fraction 2.93% and the CD45RA_PF naive fraction 2.93%, while the lowest were detected in the CD45RA_NF memory fraction 0.80% and the CD62L_PF naive 1.87% (Supplementary Table S2A,B, Figure 5F). Overall, Treg frequencies were increased in the CD45RA_NF memory fraction in both donor categories, consistent with the observed enrichment of CD4^+^ T cells in this fraction. Therefore, the CD45RA_NF memory fraction might confer some protection against GvHD. The described role of γδ T cells in anti-CMV effector functions did not correlate with the T-cell responses observed in this experiment [47].

### 2.5. Confirmation of CMV-Specific T-Cell Responses by Intracellular IFN-γ and Tumor Necrosis Factor-α (TNF-α) Cytokine Staining

**Figure 6:**
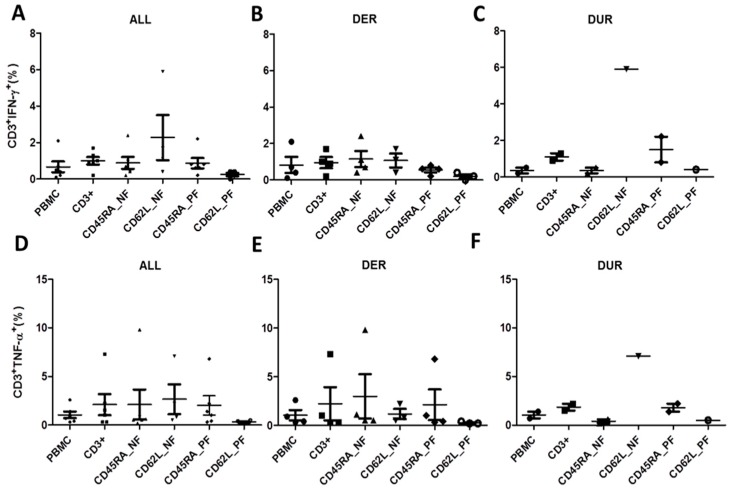
T-cell responses to ppCMV_pp65 as determined using intracellular cytokine staining (ICS). T-cell functionality depicted by IFN-γ secretion for (**A**) all donors (ALL), (**B**) donors with expected response (DER) and (**C**) donors with unexpected response (DUR) as well as TNF-α secretion for (**D**) all donors, (**E**) DER, and (**F**) DUR. Brefeldin A was added after 1 h of stimulation and incubated for 16 h. Cells were permeabilized and co-stained using antibodies against surface markers and IFN-γ and TNF-α. At least 30,000 events were measured within the lymphocyte gate by flow cytometry. Data represent the mean ± SD of *n* = 12 donors.

Donor response classification (DER vs. DUR) was further confirmed by intracellular staining for IFN-γ and TNF-α following stimulation with ppCMV_pp65 (Figure 6).

Quantification of IFN-γ and TNF-α secretion levels CD4^+^ and CD8^+^ T-cell subsets is important since it has been reported that control of CMV viremia and transfer of CMV immunity is mainly mediated by CD8^+^ T cells. Overall, TNF-α secretion levels were higher than IFN-γ secretion levels in all subsets. As expected (Figure 2A,D), we saw only slight differences in CD3^+^IFN-γ^+^ and CD3^+^TNF-α^+^ T-cell responses in all donors when comparing the CD45RA_NF and CD45RA_PF memory and naive fractions (Figure 6A,D). The highest frequencies of CD3^+^IFN-γ^+^ and CD3^+^TNF-α^+^ were found in the memory fractions of CD62L_NF 2.15% and 2.65% respectively, while the lowest were found in the naïve CD62L_PF 0.18% and 0.33% respectively (Table 3, Figure 6A,D).

**Figure 7:**
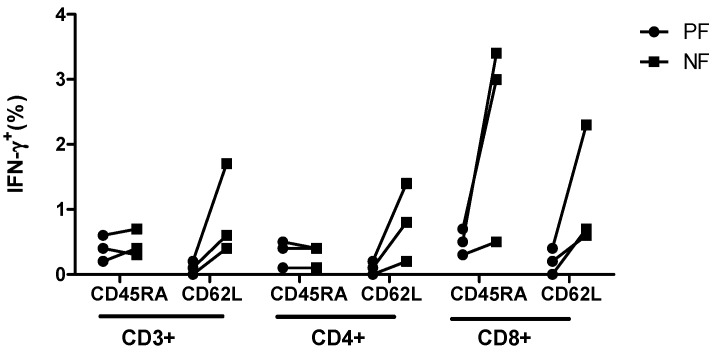
T-cell responses to ppCMV_IE1 using intracellular staining among the different T-cell fractions and subsets. After stimulation with ppCMV_IE1, the amounts of IFN-γ in the different T-cell fractions and subsets were determined by ICS. 5 × 10^5^ T cells were stimulated with 1 µg/mL ppCMV_IE1. Cells were stimulated with 50 ng/mL phorbol-12-myristate-13-acetate (PMA) and 500 ng/mL ionomycin served as positive controls and unstimulated cells served as the negative control. After 1 h of stimulation, Brefeldin A and/or monensin was added and incubated for 16 h. Cells were permeabilized and co-stained using antibodies against surface markers and IFN-γ. At least 30,000 events were acquired for each analysis. T-cell populations were gated based on the light scatter properties of lymphocytes and on IFN-γ^+^CD3^+^, IFN-γ^+^CD8^+^, and IFN-γ^+^CD4^+^. Data represent mean of three donors. NF: negative fraction (memory); PF: positive fraction (naive)

In separate analysis of DER, the ppCMV_pp65 T-cell responses depicted by IFN-γ and TNF-α were as expected (Table 3B, Figure 6). The DER/DUR classification status did not influence the outcome of the secretion analysis of IFN-γ and TNF-α in the CD62L_NF/PF memory and naive fractions (Figure 6B,C,E,F). These results clearly indicate that the observed CMV-specific T-cell response, as depicted by the IFN-γ and TNF-α frequencies, was mainly mediated by a function of CD8^+^ T cells, which secreted higher amounts of cytokines than CD4^+^ T cells. In addition, our ppCMV_IE1 stimulation experiments confirmed this pattern of T-cell response (Figure 7), and the validity of classifying the donors as DER or DUR based on their CD45RA_NF/PF memory and naive fractions was corroborated by intracellular staining.

## 3. Discussion

In immunocompromised transplant recipients, long-term control of viral infection and reactivation relies on the recovery and reconstitution of antiviral CD4^+^ and CD8^+^ T cells. Donor lymphocyte infusions (DLIs) and the transfer of enriched antiviral T cells have been proven effective in reconstituting the immune system and in preventing and controlling the reactivation of opportunistic infections following transplantation [1,20,21,22,23]. DLIs have a broad antiviral T-cell repertoire, but their application is limited because alloreactive naive T cells still present in these products have the potential to cause GvHD. The administration of enriched virus-specific memory T cells after in vitro stimulation based on IFN-γ secretion and pMHC multimers [16,20,21,22,23] has shown promising success. However, such VSTs cannot be generated if the immunodominant epitopes of the target virus (e.g., HHV6) is unknown or undefined. Thus, alternative strategies to generate clinical-grade antiviral T-cell products are needed. Therefore, this study was designed to investigate whether naive T-cell depletion using immunomagnetic CD45RA and CD62L microbeads resulted in highly pure and effective memory T-cell products (CD45RA_NF and CD62L_NF memory fractions). This research question was addressed by analyzing the phenotype compositions, T-cell frequencies and functionality of the memory T-cell fraction and the corresponding CD45RA_PF and CD62L_PF naive fractions. This article provides the first comprehensive data on human CD4^+^ and CD8^+^ T-cell responses against CMV as a virus model following naive T-cell depletion.

### 3.1. Dynamics of Sequential Cellular Composition within the Memory and Naive T-Cell Fractions

Naive T-cell depletion using CD45RA and CD62L produced two memory T-cell fractions CD45RA_NF (T_EM_ and T_CM_) and CD62L_NF (T_EM_ and T_EMRA_) and two naive T-cell fractions CD45RA_PF (T_N_ and T_EMRA_) and CD62L_PF (T_N_ and T_CM_). T_N_ depletion from starting fraction of CD3^+^ yielded a mean of 67.19% CD45RA^−^ T cells in the memory CD45RA_NF and 85.30% CD62L^−^ T cells in the memory CD62L_NF from starting fraction of CD3^+^. This is considerably higher than the yields of 47.1% and 43% obtained from PBMCs by Teschner et al. and Verfuerth et al., respectively [30,31]. These findings also confirm our hypothesis that T_N_ depletion from a starting fraction of CD3^+^ T cells produces higher yields than T_N_ depletion from PBMCs. Similar to other studies, CD45RA and CD62L depletion led to the retention of relevant memory T cells in the naive T-cell fractions [25,26,27,28,30,31]. The CD45RA_NF memory fraction contained substantial amounts of T_EMRA_ and the CD62L_PF naive fraction contained T_CM_, resulting in inter-individual variability in the magnitude of CMV-specific T-cell response.

Since the CD4^+^ T_N_ cell compartment was reported to be mainly responsible for GvHD [48], we analyzed the residual frequencies of potential alloreactive T_N_ within the CD45RA_NF and CD62L_NF memory fractions and found that strong depletion of these cells was indeed achieved. Interestingly, the depleted CD45RA_NF and CD62L_NF memory fractions contained smaller amounts of CD4^+^ T_N_ (0.33% and 0.44%) and slightly higher amounts of CD8^+^ T_N_ (2.07% and 0.92%), respectively. These findings show that both CD45RA and CD62L achieved sufficient depletion of alloreactive CD4^+^ T_N_ in comparison to 43.85% found in the PBMCs and 43.77% in the starting fraction of CD3^+^ T cells. 

Consistent to a study by Teschner et al., [30], we found that CD45RA depletion resulted in parallel depletion of CD8^+^ T cells and enrichment of CD4^+^ T cells in the CD45RA_NF memory fraction, while a slight increase in CD8^+^ T cells occurred in the CD62L_NF memory fraction. An increase in Tregs was observed as a consequence of enrichment of CD4^+^ T cells in the CD45RA_NF memory fraction [30]. Tregs are known to be potent suppressors of immune responses that regulate alloreactive donor T-cell responses and, thus, play a role in mitigating the severity of GvHD [49]. It is therefore postulated that Tregs in the CD45RA_NF memory fraction could confer some additional protection against GvHD [44]. On the down side, Tregs are reported to be involved the suppression of antiviral immune responses and thus may impair successful CMV-specific immune response by the CD45RA_NF memory fraction [49]. Furthermore, the lowest Treg frequencies occurred in the CD62L_NF memory fraction. This could be explained by the fact that CD62L depletion results in decreased amounts of CD4^+^ T cells. On the other hand, compared to the starting fraction, γδ T-cell frequencies increased in all T-cell fractions with exception of CD45RA_NF memory fraction. This is in line with a study by Mueller et al., who also observed a decrease in γδ T cells within the CD45RA_NF memory fraction [50]. γδ T cells have been described as important immune effectors against CMV in vivo [47], the results of the present study do not clearly confirm that they play such a role in vitro.

### 3.2. Dynamics of Sequential Cellular Composition within the Memory and Naive T-Cell Fractions

The phenotypic characteristics of virus-specific T cells in different chronic infections vary due to different patterns of response. Furthermore, associations between the functional attributes of CD4^+^ and CD8^+^ antiviral T cells against different pathogens contribute to the overall antiviral immune responses [34,39]. Utilizing tetramer analysis to detect antiviral T cells, Appay and co-workers showed that 50–60% of CMV_pp65-specific T cells were present in the CD8^+^ T_EMRA_ population, while EBV-multimer-positive T cells (80–90%) predominated in the CD8^+^CD45RO^+^CD27^+^ (memory) T-cell fraction [38]. This suggests that CMV_pp65-specific T-cell response could be mainly due to CD8^+^ T_EMRA_. In our analysis, CMV-specific T-cell responses were higher overall in the memory fractions than in the respective naive fractions, in line with previous reports [26,30,31]. However, our in-depth analysis showed variability in the responses observed for the CD45RA_NF and CD45RA_PF memory and naive fractions, prompting us to classify donors according to expected response (DER) and unexpected response (DUR). 50% of the donors had higher ppCMV_pp65 T-cell responses in the CD45RA_PF naive fraction and were thus classified as DUR. Our extensive phenotypic analysis revealed that, compared to DER, the DUR had higher frequencies of CD8^+^ T_EMRA_ observed in PBMCs that were further enriched within the CD45RA_PF naive fraction following naïve T-cell depletion.

We also observed stronger CMV-specific responses in CD8^+^IFN-γ^+^ and CD8^+^TNF-α^+^ in all memory T-cell fractions, consistent with previous reports [30,31]. However, we detected inverse IFN-γ^+^ and TNF-α^+^ secretion responses in CD4^+^ subsets in both the memory and the naive T-cell fraction that were lower than those in the corresponding CD3^+^ and CD8^+^ T-cell subsets (Table 3A). Despite the high frequencies of T_CM_ in the CD62L_PF naive fraction, the lowest CMV-specific T-cell responses were obtained there. In-depth analysis showed that most of those T_CM_ cells were in the CD4^+^ subsets. This could be due to the fact that direct anti-CMV-specific T-cell responses are mainly mediated by a function of CD8^+^ T cells during periods of high viremia. The fact that CD4^+^ T cells support the state of antiviral immunity during persistent infection [51] might explain the lower responses within the CD62L_PF naive fraction. Nonetheless, it is reported that the enrichment of CD4^+^ T cells in the CD45RA_NF memory fraction might improve responses to viruses which are mainly mediated by CD4^+^ T helper cells, e.g., in the case of adenovirus [40] and Dengue virus [41]. 

Based on the observed variability of CMV-specific T-cell responses in the CD45RA fractions, we hypothesize that this phenomenon could be a consequence of CD45RA depletion of CD8^+^ T_EMRA_ in the CD45RA_NF memory fraction. Various studies assert that the CD8^+^ T_EMRA_ population provides superior clearance of CMV infection as is highly cytotoxic and has high avidity [52], expressing effector cytokines such as IFN-γ and TNF-α [53,54]. A clinical study by Luo and colleagues elegantly showed that allografts consisting of the T_EMRA_ phenotype were associated with a reduced risk of CMV reactivation [55]. Furthermore, they suggested that as T_EM_ frequencies are just as important as T_EMRA_ frequencies, their depletion potentially decreases the overall magnitude of anti-CMV T-cell responses [30]. Taken together, our findings demonstrate that CD8^+^ T_EMRA_ and T_EM_ could contribute immensely to the overall efficacy of the anti-CMV response capacity, as shown by the CD62L_NF memory fraction. We showed that the CMV T-cell responses in the naive CD45RA_PF among the unexpected responders (DUR) were also mediated by a function of CD8^+^ T_EMRA_. For this reason, T_EMRA_ should be considered in patients with active CMV infection post-HSCT although they will only provide transient protection in the recipient due to their half-life of approximately 45 to 60 days [56,57]. An additional advantage is that they transiently re-express CCR7, lymph node homing receptor and CXC chemokine receptor 3 (CXCR3) which are useful for migration into peripheral tissues and organs [53,58] that could help virus-specific T cells to infiltrate the affected organs.

### 3.3. Dichotomy of CD45RA and CD62L Naive T-Cell Depletion Characteristics

Based on the high CMV-specific responses consistently observed in all donors, the results of our comparison of CD45RA and CD62L depletion strategies suggest that T_N_ depletion using CD62L offers substantial benefits over CD45RA with respect to CMV. Central memory T cells have been reported to potentially trigger the development of GvHD [59] due to cross reactivity to alloantigens. Thus, CD45RA depletion may be less advantageous in preventing GvHD than CD62L depletion. Nonetheless, CD8^+^ T_CM_ might be necessary for long-term memory and engraftment [60]. Scheinberg et al. reported that adoptively transferred CMV-specific T-cell clones derived from the T_CM_ pool are more persistent in recipient macaques than effector cells [57]. Furthermore, central memory T cells have the capacity to expand and differentiate upon challenge. Consequently, it is reasonable to assume that T_EMRA_ and T_EM_ will be reconstituted from T_CM_ cells in patients in vivo after infusion [35]. Therefore, these factors should be considered when selecting a depletion strategy. A drawback of CD62L depletion is that granulocyte-colony stimulating factor (GCSF) may cause shedding off due to proteolytic cleavage [61] and, therefore, T_N_ might not be efficiently depleted if DLIs are collected from the stem cell donor.

### 3.4. Paradigm Shift in the Selection of T-Cell Products in Clinical Practice

Various clinical trials only considered the CD45RA_NF subset for immunotherapy and, thus, might not have fully utilized the anti-viral T cells present in the donor [26,30,31,50]. Our data show that CMV-specific T cells have a restricted functional profile that is in large part determined by the donor’s T-cell phenotypes. The quantity and quality of CMV-specific CD8^+^ T_EMRA_ are expected to correlate with the overall potency of the response that will be obtained after infusion. In case of CMV infection, CD62L microbeads should provide effective products due to the presence of CD8^+^ T_EMRA_ and T_EM_ in this fraction. Our analysis showed that CD62L depletion also provided a higher yield of 85.30% (cell yield of 6.30 × 10^6^ from a starting population of 7.51 × 10^6^ memory CD62L^−^ T cells) than that obtained by CD45RA depletion, which yielded 67.19% (cell yield of 7.34 × 10^6^ from a starting population of 10.99 × 10^6^ memory CD45RA^−^ T cells). Regarding the cell numbers required for adoptive T-cell transfer, a study by Luo showed that 0.177 × 10^6^/kg of T_EMRA_ when infused with sufficient amounts of T_EM_ (≥0.208 × 10^6^/kg) in donor allografts was associated with a reduced risk of CMV reactivation [55]. It is logically possible to assert that CMV-seropositive donors with an expanded population of memory T cells such as CD8^+^ T_EMRA_ as well as substantial amounts of T_EM_ will be optimal. Our analysis clearly showed that DUR had more T_EMRA_ within the PBMCs in the CD8^+^ T-cell subset and hence have higher CMV-specific T-cell responses in the CD45RA_PF naive fraction. This indicates that T-cell responses induced in the recipient mirror those in the donor in relation to the phenotypic composition, providing a window of possibility for predicting the donor classification and the most efficient T_N_ depletion method. Paradoxically, an increase in the T_EMRA_ population occurred in parallel to a decrease in T_CM_ seen in PBMC and CD3^+^ T-cell fraction among CD3^+^ versus CD8^+^ T-cell subset. Accordingly, in order to retain T_EMRA_, CD62L microbeads should be preferentially selected because the amount of T_CM_ consequentially lost in the CD8^+^ T-cell subset is not substantial. This prediction could also be applicable to viruses known to establish latency in the hosts and reported to have sustained T_EMRA_, e.g., parvoviruses (B19 and PARV49), herpes simplex virus 1 (HSV-1) [52].Thus, CD45RA depletion will provide optimal benefit in the case of viral infections that rely on CD4^+^ T-cell function. Therefore, extensive phenotyping and detection of antiviral T-cell frequencies from recollection samples can be used as a relevant immunodiagnostic strategy to predict the magnitude of T-cell depletion for clinical applications.

## 4. Materials and Methods 

### 4.1. Isolation of PBMCs and T Cells

Experiments were performed with residual blood samples from platelet apheresis disposable kits used for routine platelet collection from regular healthy blood donors of the Hannover Medical School (MHH) Institute for Transfusion Medicine. Informed consent as approved by the Ethics Committee of Medical School Hannover was obtained from all donors (ethics votum: 3639-2017 on 14 December 2017). Peripheral blood mononuclear cells (PBMCs) were isolated from CMV-seropositive donors by discontinuous-gradient centrifugation. Untouched CD3^+^ T cells were enriched by magnetic cell sorting (MACS) using Pan T-cell isolation kit (Miltenyi Biotec, Bergisch Gladbach, Germany), according to the manufacturer`s instructions. CD3^+^ T cells were collected as flow through and bead-loaded non-T cells were collected from columns as eluate and were further used as target cell population in the target-cell dependent ELISpot assay. Further, the isolated CD3^+^ T cells were used for naïve T-cell depletion using CD45RA or CD62L immunomagnetic microbeads (Miltenyi Biotec). Negative fractions (flow-through) and positive fractions (eluate) were collected by LS^+^ and or LD^−^ columns (both Miltenyi Biotech) and termed as follows: memory CD3^+^CD45RA^−^ (CD45RA_NF) and CD3^+^CD62L^−^ (CD62L_NF) fractions and the naïve fractions CD3^+^CD45RA^+^ (CD45RA_PF) and CD3^+^CD62L^+^ (CD62L_PF).

### 4.2. Flow Cytometry Analysis

For phenotypic characterization, 3 × 10^5^ cells were stained with fluorescein-isothiocyanate (FITC) anti-CD3, peridinin chlorophyll (PerCP)-conjugated anti-CD4, alexa fluor (AF) 700-conjugated CD8, brilliant violet (BV) 510-conjugated anti-CD45RA, allophycocyanin/Cyanin 7 (APC/Cy7)-conjugated anti-CD62L, anti-CD45RO-phycoerythrin (PE), and AF647-conjugated anti-CD197 (all BioLegend, London, Great Britian) monoclonal antibodies for 20 min at room temperature in the dark, washed with PBS (Lonza, Verviers, Belgium) with 0.1% human AB serum (C.C. pro, Oberdorla, Germany) and analyzed by multicolor flow cytometry (FACS Canto II, FACSDiva V8.1.2 software, BD Biosciences, Heidelberg, Germany). Gates were set based on the forward scatter versus side scatter properties of lymphocytes. At least 30,000 events were acquired in the CD3^+^ gate. For detailed gating strategy see Supplementary Figure S1B,C. For intracellular staining (ICS) peridinin chlorophyll (PerCP)-conjugated anti-CD3, alexa fluor (AF) 700-conjugated CD8, additional fluorescein isothiocyanate (FITC)-conjugated anti-IFN-γ and APC-conjugated TNF-α antibodies were used (both BioLegend). To determine Tregs and γδ T cells, alexa fluor (AF) 700-conjugated CD4, BV421-conjugated anti-CD25 (BioLegend), APC-conjugated anti-CD127, and anti-γδ TCR-PE/Cy7 antibodies were used (both BD Biosciences).

### 4.3. CMV-Specific T-Cell Response Determined by IFN-γ ELISpot

Functionality of the different T-cell fractions were analyzed by IFN-γ ELISpot assay with (target cell-dependent) and without target cells (target cell-independent). The target cell-independent assay was performed as described previously [62,63]. Briefly, PBMCs and the different T-cell fractions were isolated on day 0 and rested overnight in T-cell culture medium Roswell Park Memorial Institute medium 1640 (RPMI1640) (Lonza, Vervies, Belgium) at 1 × 10^7^ cells per ml/well with 10% heat-inactivated human AB serum (TCM) (C.C.pro, Oberdorla, Germany) in a 24 well tissue culture plate (Sarstedt, Nümbrecht, Germany) at 37 °C and 5% CO_2_. On day 1, cells were resuspended with fresh medium, counted and seeded at a concentration of 2.5 × 10^5^ PBMCs as well as 5 × 10^4^ effector T cells cells/well in a 96 well plate (pre-coated anti-IFN-γ ELISpot plate; Lophius Biosciences, Regensburg, Germany) and overnight stimulation with 1 µg/mL 15-mer overlapping peptide pools (pp) of ppCMV_pp65 or ppCMV_IE1 (JPT Peptide Technologies, Berlin Germany). The target cell-dependent assay utilized target cells (CD3 negative cells), rested on day 0 in TexMACS medium (Miltenyi Biotec, Germany) and seeded at a density of 1×10^7^ cells per mL/well. Target cells were then stimulated overnight with 1 µg/mL ppCMV_pp65 or ppCMV_IE1 per 1×10^7^/mL. On day 1, the T-cell fractions (effector cells) were seeded at a concentration of 5×10^4^ T effector cells with the target cells in effector: target (E.T) ratios of 1:1 and or 2:1 in IFN-γ ELISpot plate. Untreated cells served as negative control and for positive control, cells were stimulated with 1µg/mL SEB (staphylococcus enterotoxin B) (Sigma-Aldrich, Hamburg, Germany) diluted in TCM. IFN-γ secretion was detected following 16 h of incubation at 37 °C and 5% CO_2_ using biotin-conjugated antihuman IFN-γ antibodies (mAb 7-B6-1-biotin, Mabtech, Stockholm, Sweden) and streptavidin-alkaline phosphatase (Mabtech) revealed by 5-bromo-4-chloro-3-indolyl phosphate/nitroblue tetrazolium (BCIP/NBT Liquid Substrate, Sigma-Aldrich, Germany). The data were acquired on an ‘AID iSpot Reader System’ (AID GmbH, Straßberg, Germany) with ‘AID ELISpot Software Version 7.0′ (https://www.aid-diagnostika.com) and spot counting was performed with ‘AID ELISpot Software Version 8.0′ (https://www.aid-diagnostika.com). All spot counts are mean values of duplicate wells and expressed as spot-forming unit per well per 100,000 CD3^+^ T cells (spwT). The cut-off for positive response was at least two times higher than the negative control. All spot counts were mean values from duplicate wells.

### 4.4. Detection of IFN-γ and TNF-α by Intracellular Cytokine Staining

Freshly isolated T-cell fractions (effector cells) were rested in a 24 well plate in TCM overnight (1 × 10^7^ cells per mL/well). On day 1, 5 × 10^5^ effector cells/ 200 µL in RPMI were seeded in a 96 well U-bottom plate (BD Biosciences, Heidelberg, Germany) and stimulated with 1 µg/mL ppCMV_pp65 or ppCMV_IE1. As a positive control, cells were stimulated with 50 ng/mL PMA and 500 ng/mL Ionomycin and as negative controls; only culture medium and effector cells without stimulation were used. The plates were incubated at 5% CO_2_ and 37 °C for 60 min. Brefeldin A (1 μg/mL, BD Biosciences, Heidelberg, Germany) and/or Monensin (10 μg/mL, MerckKGaA, Darmstadt, Germany) was added and the cells were incubated for 16 h. Cells were then permeabilized with IntraPrep (Beckman Coulter, Brea, CA, USA), stained with respective antibodies and analyzed by flow cytometry. At least 30,000 events were acquired and gated based on the light scatter properties of lymphocytes followed by IFN-γ^+^CD3^+^, IFN-γ^+^CD8^+^, and IFN-γ^+^CD4^+^ T-cell populations.

### 4.5. Statistical Analysis

Statistical analyses were performed in GraphPad Prism version 7.0 software (GraphPad Software, San Diego, CA, USA) using unpaired Student’s *t*-test and Kruskal–Wallis test. Levels of significance were expressed as *p*-values (* *p* < 0·05, ** *p* < 0·01, *** *p* < 0·001, **** *p* < 0·0001, not significant (n.s.)).

## 5. Conclusions

Based on the results of this study, we propose to determine the phenotypic composition and consequentially the best depletion strategy to retain the predominant T cells when selecting T-cell isolation strategies and donors. Alternatively, a one-step depletion method using antibody-conjugated microbeads that only targets T_N_ cells or a two-step combination depletion method using reversible staining protocols for T_N_ depletion should be established in order to salvage the important T-cell populations that are currently depleted by the conventional methods.

## Figures and Tables

**Table 1:** Assessment of T-cell frequencies, phenotypes and cellular composition among all donors. T-cell frequencies and phenotypes in different T-cell fractions, as determined by flow cytometry (mean, range; *n* = 24) in naive T-cell depleted samples. T-cell frequencies are expressed as mean % of CD3^+^, CD4^+^, and CD8^+^ T cells. Tregs (CD4^+^ CD25^+^ CD127^low^) were gated among CD4^+^ T cells (*n* = 8 donors) and γδ T cells gated among CD3^+^ T cells in the different T-cell subsets (*n* = 8 donors).

T-Cell Subset	PBMCs	CD3+	CD45RA_NF	CD62L_NF	CD45RA_PF	CD62L_PF
**CD3+ [%]**	52.39 (32.7–73.8)	96.35 (91.9–98.9)	98.08 (95.3–99.7)	96.19 (91.2–98.5)	94.83 (86.8–99.0)	96.11 (90.4–98.9)
T_N_ [%]	43.46 (19.6–63.2)	43.6 (14.7–65.8)	1.06 (0–8.3)	1.63 (0.0–13.0)	65.97 (35.0–91.9)	54.78 (32.2–74.5)
T_CM_ [%]	28.62 (153–49.0)	25.13 (10.6–44.8)	56.99 (31.4–74.2)	1.77 (0.0–15.4)	1.08 (0.2–4.6)	41.18 (22.9–67.0)
T_EM_ [%]	19.18 (9.3–34.7)	20.4 (8.0–40.8)	40.77 (24.0–60.4)	66.15 (32.7–90.3)	1.1 (0.2–4.0)	2.6 (0.2–9.4)
T_EMRA_ [%]	9.72 (1.4–28.1)	11.57 (17–32.7)	1.11 (0.0–17.8)	28.7 (7.1–64.5)	33.39 (7.2–62.6)	1.15 (0.0–6.3)
**CD4+ [%]**	77.97 (51.3–93.2)	68.28 (46.7–87.7)	82.81 (71.3–92.6)	48.98 (31.9–81.3)	59.84 (36.8–86.6)	73.99 (53.4–94.1)
**CD8+ [%]**	18.85 (3.4–48.7)	30.29 (8.9–50.3)	16.34 (7.2–27.9)	48.47 (16.1–63.9)	38.15 (9.8–63.2)	24.66 (3.6–46.6)
**CD4/8 ratio**	4.13	2.25	5.08	1.01	1.56	3.0
**Tregs [%]**	5.7 (3.1–9.3)	5.8 (2.7–11.5)	6.58 (1.2–13.4)	4.85 (1.1–12.1)	5.1 (1.7–9.7)	5.13 (2.5–7.4)
**γδ T cells [%]**	3.16 (0.5–6.1)	1.9 (0.5–3.7)	1.41 (0.3–4.0)	2.63 (0.6–5.7)	2.7 (0.5–4.3)	2.02 (0.5–5.7)

**Table 2:** Phenotype, T-cell counts and cellular composition in donors with expected responses (DER) and unexpected responses (DUR). T-cell frequencies and phenotypes in different T-cell fractions, as determined by flow cytometry (mean, range; *n* = 12) in naive T-cell depleted samples. T-cell frequencies are expressed as mean % of CD3^+^, CD4^+^and CD8^+^ T cells.

T-Cell Subset	PBMC	CD3+	CD45RA_NF	CD62L_NF	CD45RA_PF	CD62L_PF
**CD3+ [%]**	54.52 (32.7–73.8)	96.15 (92.4–98.4)	98.25 (96.7–99.7)	96.08 (92.0–98.0)	94.48 (88.3–98.7)	95.79 (90.4–98.8)
T_N_ [%]	43.85 (19.6–63.2)	43.77 (23–65.8)	0.88 (0.0–8.3)	0.9 (0.0–6.8)	67.28 (35–91.9)	59.49 (32.2–74.5)
T_CM_ [%]	26.70 (15.3–49.0)	22.96 (10.6–44.8)	54.08 (31.4–74.2)	0.82 (0.0–8.6)	0.57 (0.2–1.1)	38.72 (25.3–67)
T_EM_ [%]	19.52 (9.3–30)	21.05 (10.9–31.7)	43.48 (25.6–58.2)	65.08 (34.5–89.3)	0.78 (0.3–1.8)	1.042 (0.2–3.1)
T_EMRA_ [%]	9.93 (1.4–28.1)	12.24 (3.1–32.7)	1.55 (0.0–17.8)	33.17 (10.6–64.5)	31.37 (7.2–65.6)	0.76 (0–2.7)
**CD4+ [%]**	77.21 (51.3–93.2)	65.41 (51.3–82.9)	80.58 (74.8–90.7)	44.49 (32.6–56.4)	56.86 (36.8–76.1)	71.98 (53.4–89.2)
T_N_ [%]	44.08 (13.6–60.8)	42.11 (13–63)	0.33 (0.0–1.8)	2.07 (0.0–11.0)	83.78 (64.2–96.7)	52.7 (17.2–72.7)
T_CM_ [%]	33.76 (21.1–72.0)	33.02 (16.6–59.7)	59.97 (44.9–74.3)	8.42 (0–77.8)	1.13 (0.1–3.3)	45.72 (27.1–82.4)
T_EM_ [%]	17.40 (10.9–35.8)	20.1 (10.0–35.3)	39.51 (25.5–55)	77.29 (11.5–98.8)	1.05 (0.2–2.6)	1.275 (0.2–4)
T_EMRA_ [%]	4.77 (0.4–20.8)	4.2 (0.2–21.2)	0.19 (0.0–1.6)	12.23 (0.6–45.2)	14.03 (2.5–33.6)	0.29 (0.0–1.0)
**CD8+ [%]**	20.08 (6.3–48.7)	33.39 (15.9–48.7)	18.80 (9.0–25.2)	53.12 (42.0–63.0)	41.31 (22.1–63.2)	27.03 (9.9–46.6)
T_N_ [%]	35.7 (0.8–81.9)	39.63 (22.1–80.8)	0.44 (0.1–1.5)	0.92 (0.0–3.6)	42.23 (17.4–89.6)	66.41 (34.3–93.7)
T_CM_ [%]	7.99 (0.2–25.8)	10.26 (1.8–35.7)	31.57 (13.3–62.8)	0.43 (0.0–4.0)	0.88 (0.1–6.2)	27.78 (2.8–60.9)
T_EM_ [%]	25.73 (3.2–51.5)	29.96 (2.9–48.8)	67.58 (36.6–86.2)	49.33 (7.1–77.7)	0.94 (0.1–2.1)	3.47 (0.3–13.3)
T_EMRA_ [%]	30.58 (7.4–72.1)	20.16 (3.1–51)	0.39 (0.0–1.5)	49.29 (20.3–91.2)	55.94 (9.6–81.7)	2.33 (0.1–6.4)
**CD4/8 ratio**	3.85	1.96	4.29	0.84	1.38	2.66
**Tregs [%]**	6.43 (3.3–9.3)	6.63 (4.8–11.5)	7.62 (1.2–13.4)	4.42 (1.1–12.1)	5.7 (1.7–9.7)	5.57 (2.5–7.4)
**γδ T cells [%]**	2.93 (0.5–4.6)	1.73 (0.5–3.2)	1.16 (0.3–2.9)	2.22 (0.6–4.30)	2.68 (0.5–3.8)	2.3 (0.5–5.7)

**Table 3:** T-cell responses to ppCMV_IE1 using intracellular staining within the different T-cell fractions and subsets (**A**) Frequencies of IFN-γ^+^CD3^+^, IFN-γ^+^CD4^+^ and IFN-γ^+^CD8^+^ secretion as well as TNF-α^+^CD3^+^, TNF-α^+^CD4^+^, and TNF-α^+^CD8^+^ within the different T-cell fractions among all donors (ALL); (**B**) among donors with expected response (DER); and (**C**) among donors with unexpected response (DUR).

**A**						
**ppCMV_pp65 (ALL)**						
**IFN-γ**	**PBMCs**	**CD3+**	**CD45RA_NF**	**CD62L_NF**	**CD45RA_PF**	**CD62L_PF**
CD3+IFN-γ+	0.28 (0.1–0.5)	0.72 (0.2–1.3)	0.42 (0.2–0.7)	2.15 (0.4–5.9)	0.84 (0.2–2.2)	0.18 (0.0–0.4)
CD4+IFN-γ+	0.26 (0.1–0.6)	0.3 (0.1–0.6)	0.2 (0.0–0.4)	1.33 (0.2–2.9)	0.44 (0.1–0.8)	0.13 (0.0–0.2)
CD8+IFN-γ+	0.54 (0.2–1.0)	1.44 (0.3–2.8)	1.82 (0.3–3.4)	3 (0.6–8.4)	1.38 (0.3–3.4)	0.38 (0.0–0.9)
**TNF-ɑ**	**PBMCs**	**CD3+**	**CD45RA_NF**	**CD62L_NF**	**CD45RA_PF**	**CD62L_PF**
CD3+TNF-ɑ+	0.74 (0.7–1.4)	1.06 (0.3–2.2)	0.58 (0.2–1.1)	2.65 (0.5–7.1)	1.06 (0.3–2.2)	0.33 (0.2–0.5)
CD4+TNF-ɑ+	0.76 (0.2–2.0)	0.66 (0.2–1.3)	0.3 (0.1–0.7)	1.73 (0.4–3.6)	0.52 (0.3–1.0)	0.23 (0.1–0.4)
CD8+TNF-ɑ+	1.04 (0.7–1.4)	2.02 (0.8–3.9)	2.3 (0.7–3.8)	3.35 (0.9–9.0)	1.76 (0.5–3.5)	0.63 (0.3–1.2)
**ppCMV_IE1 (ALL)**						
**IFN-γ**	**PBMCs**	**CD3+**	**CD45RA_NF**	**CD62L_NF**	**CD45RA_PF**	**CD62L_PF**
CD3+IFN-γ+	0.26 (0.1–0.6)	0.64 (0.1–2.0)	0.36 (0.1–0.9)	0.45 (0.0–1.5)	1.13 (0.0–5.8)	0.13 (0.0–0.3)
CD4+IFN-γ+	0.20 (0.0–0.7)	0.18 (0.0–0.5)	0.06 (0.0–0.1)	0.08 (0.0–0.1)	0.64 (0.0–2.5)	0.05 (0.0–0.1)
CD8+IFN-γ+	0.38 (0.1–0.8)	1.18 (0.1–3.7)	1.42 (0.1–4.1)	0.93 (0.0–3.2)	2.16 (0.0–9.1)	0.25 (0.0–0.7)
**TNF-ɑ**	**PBMCs**	**CD3+**	**CD45RA_NF**	**CD62L_NF**	**CD45RA_PF**	**CD62L_PF**
CD3+TNF-ɑ+	0.66 (0.2–1.0)	1.06 (0.1–3.3)	0.52 (0.1–1.3)	3.05 (0.3–7.3)	1.6 (0.2–6.2)	0.23 (0.1–0.5)
CD4+TNF-ɑ+	0.62 (0.1–2.0)	0.52 (0.1–1.3)	0.14 (0.0–0.2)	0.23 (0.1–0.4)	0.76 (0.1–2.1)	0.13 (0.1–0.2)
CD8+TNF-ɑ+	1.06 (0.5–1.9)	2.04 (0.5–5.5)	2.3 (0.3–6.0)	4.88 (0.6–11.4)	2.84 (0.3–10.1)	0.45 (0.2–1.0)
**B**						
**ppCMV_pp65 in donors with expected response (DER)**						
**IFN-γ**	**PBMCs**	**CD3+**	**CD45RA_NF**	**CD62L_NF**	**CD45RA_PF**	**CD62L_PF**
CD3+IFN-γ+	0.23 (0.1–0.4)	0.47 (0.2–0.9)	0.47 (0.3–0.7)	0.9 (0.4–1.7)	0.4 (0.2–0.6)	0.1 (0.0–0.2)
CD4+IFN-γ+	0.17 (0.1–0.2)	0.2 (0.1–0.4)	0.3 (0.1–0.4)	0.8 (0.2–1.4)	0.33 (0.1–0.5)	0.1 (0.1–0.2)
CD8+IFN-γ+	0.57 (0.2–1.0)	0.97 (0.3–2.1)	2.3 (0.5–3.4)	1.2 (0.9–2.4)	0.5 (0.3–0.7)	0.2 (0.0–0.4)
**TNF-ɑ**	**PBMCs**	**CD3+**	**CD45RA_NF**	**CD62L_NF**	**CD45RA_PF**	**CD62L_PF**
CD3+TNF-ɑ+	0.53 (0.3–0.9)	0.53 (0.3–1.0)	0.7 (0.5–1.1)	1.17 (0.5–2.2)	0.57 (0.3–1.0)	0.27 (0.2–0.4)
CD4+TNF-ɑ+	0.43 (0.2–0.7)	0.33 (0.2–0.5)	0.43 (0.2–0.7)	1.1 (0.4–2)	0.53 (0.3–1)	0.2 (0.1–0.4)
CD8+TNF-ɑ+	0.93 (0.7–1.3)	1.3 (0.8–2.2)	2.67 (0.7–3.8)	1.47 (0.9–2.4)	0.83 (0.5–1.0)	0.43 (0.3–0.5)
**ppCMV_IE1 in donors with expected response (DER)**						
**IFN-γ**	**PBMCs**	**CD3+**	**CD45RA_NF**	**CD62L_NF**	**CD45RA_PF**	**CD62L_PF**
CD3+IFN-γ+	0.1 (0.1–0.1)	0.1 (0.1–0.2)	0.1 (0.1–0.4)	0.2 (0.1–0.4)	0.1 (0.0–0.2)	0.07 (0.0–0.1)
CD4+IFN-γ+	0.1 (0.1–0.2)	0.03 (0.0–0.1)	0.05 (0.0–0.12)	0.1 (0.1–0.2)	0.1 (0.0–0.1)	0.03 (0.0–0.1)
CD8+IFN-γ+	0.2 (0.1–0.3)	0.17 (0.1–0.3)	0.1 (0.1–0.1)	0.3 (0.2–0.4)	0.2 (0.1–0.3)	0.13 (0.0–0.3)
**TNF-ɑ**	**PBMCs**	**CD3+**	**CD45RA_NF**	**CD62L_NF**	**CD45RA_PF**	**CD62L_PF**
CD3+TNF-ɑ+	0.27 (0.2–0.3)	0.2 (0.1–0.3)	0.1 (0.1–0.5)	0.37 (0.3–0.5)	0.43 (0.2–0.4)	0.13 (0.1–0.2)
CD4+TNF-ɑ+	0.2 (0.1–0.4)	0.17 (0.1–0.3)	0.05 (0.2–0.3)	0.23 (0.1–0.4)	0.43 (0.2–0.3)	0.1 (0.1–0.1)
CD8+TNF-ɑ+	0.63 (0.5–0.9)	0.47 (0.4–0.5)	0.35 (0.3–0.4)	0.7 (0.6–0.8)	0.57 (0.3–0.8)	0.23 (0.2–0.3)
**C**						
**ppCMV_pp65 in donors with unexpected response (DUR)**						
**IFN-γ**	**PBMCs**	**CD3+**	**CD45RA_NF**	**CD62L_NF**	**CD45RA_PF**	**CD62L_PF**
CD3+IFN-γ+	0.35 (0.2–0.5)	1.1 (0.9–1.3)	0.35 (0.2–0.5)	5.9 (5.9–5.9)	1.5 (0.8–2.2)	0.4 (0.4–0.4)
CD4+IFN-γ+	0.4 (0.2–0.6)	0.45 (0.3–0.6)	0.05 (0.0–0.1)	2.9 (2.9–2.9)	0.6 (0.4–0.8)	0.2 (0.2–0.2)
CD8+IFN-γ+	0.5 (0.4–0.6)	2.15 (1.5–2.8)	1.1 (0.3–1.9)	8.4 (8.4–8.4)	2.7 (2.0–3.4)	0.9 (0.9–0.9)
**TNF-ɑ**	**PBMCs**	**CD3+**	**CD45RA_NF**	**CD62L_NF**	**CD45RA_PF**	**CD62L_PF**
CD3+TNF-ɑ+	1.05 (0.7–1.4)	1.85 (1.5–2.2)	0.4 (0.2–0.6)	7.1 (7.1–7.1)	1.8 (1.4–2.2)	0.5 (0.5–0.5)
CD4+TNF-ɑ+	1.25 (0.5–2.0)	1.15 (1.0–1.3)	0.1 (0.1–0.1)	3.6 (3.6–3.6)	0.5 (0.5–0.5)	0.3 (0.3–0.3)
CD8+TNF-ɑ+	1.2 (1.0–1.4)	3.1 (2.3–3.9)	1.75 (0.9–2.6)	9.0 (9.0–9.0)	3.15 (2.8–3.5)	1.2 (1.2–1.2)
**ppCMV_IE1 in donors with unexpected response (DUR)**						
**IFN-γ**	**PBMCs**	**CD3+**	**CD45RA_NF**	**CD62L_NF**	**CD45RA_PF**	**CD62L_PF**
CD3+IFN-γ+	0.37 (0.1–0.6)	1.0 (0.4–2.0)	0.53 (0.3–0.9)	0.75 (0.0–1.5)	2.17 (0.0–5.8)	0.2 (0.1–0.3)
CD4+IFN-γ+	0.27 (0.0–0.7)	0.3 (0.0–0.5)	0.07 (0.0–0.1)	0.07 (0.0–0.1)	1.03 (0.0–2.5)	0.07 (0.0–0.1)
CD8+IFN-γ+	0.53 (0.2–0.8)	1.9 (0.8–3.7)	2.3 (1.4–4.1)	1.6 (0.0–3.2)	3.5 (0.0–9.1)	0.45 (0.2–0.7)
**TNF-ɑ**	**PBMCs**	**CD3+**	**CD45RA_NF**	**CD62L_NF**	**CD45RA_PF**	**CD62L_PF**
CD3+TNF-ɑ	0.97 (0.5–1.7)	1.63 (0.7–3.3)	0.8 (0.5–1.3)	5.8 (4.3–7.3)	2.5 (0.4–6.2)	0.35 (0.2–0.5)
CD4+TNF-ɑ+	0.93 (0.4–2.0)	0.73 (0.1–1.3)	0.2 (0.2–0.2)	0.2 (0.2–0.2)	1.1 (0.1–2.1)	0.15 (0.1–0.2)
CD8+TNF-ɑ+	1.3 (0.5–1.9)	3.07 (1.8–5.5)	3.6 (2.0–6.0)	9.1 (6.8–11.4)	4.43 (1.0–10.1)	0.7 (0.4–1.0)

## Supplementary Materials

Supplementary materials can be found at https://www.mdpi.com/1422-0067/20/6/1415/s1.

## Abbreviations

DER	Donors with expected response
DUR	Donors with unexpected response
CCS	Cytokine capture system
CMV	Cytomegalovirus
EBV	Epstein–Barr virus
GCSF	Granulocyte-colony stimulating factor
GvHD	Graft-versus-host disease
HSCT	Hematopoietic stem-cell transplantation
ICS	Intracellular cytokine staining
VST	Virus-specific T cell
NF	Negative fraction
PF	Positive fraction
Treg	Regulatory T cell
