# Supplementary material for: Robust Identification of Suitable T-Cell Subsets for Personalized CMV-Specific T-Cell Immunotherapy Using CD45RA and CD62L Microbeads
Source: Int J Mol Sci. 2019 Mar 20;20(6):1415. doi: 10.3390/ijms20061415 (PMC6471767; doi:10.3390/ijms20061415)
Supplement: Supplementary file 1 [file ijms-20-01415-s001.pdf]

Robust identification of suitable T-cell subsets for personalized CMV-specific T-cell immunotherapy using CD45RA and CD62L microbeads

Supplementary figures

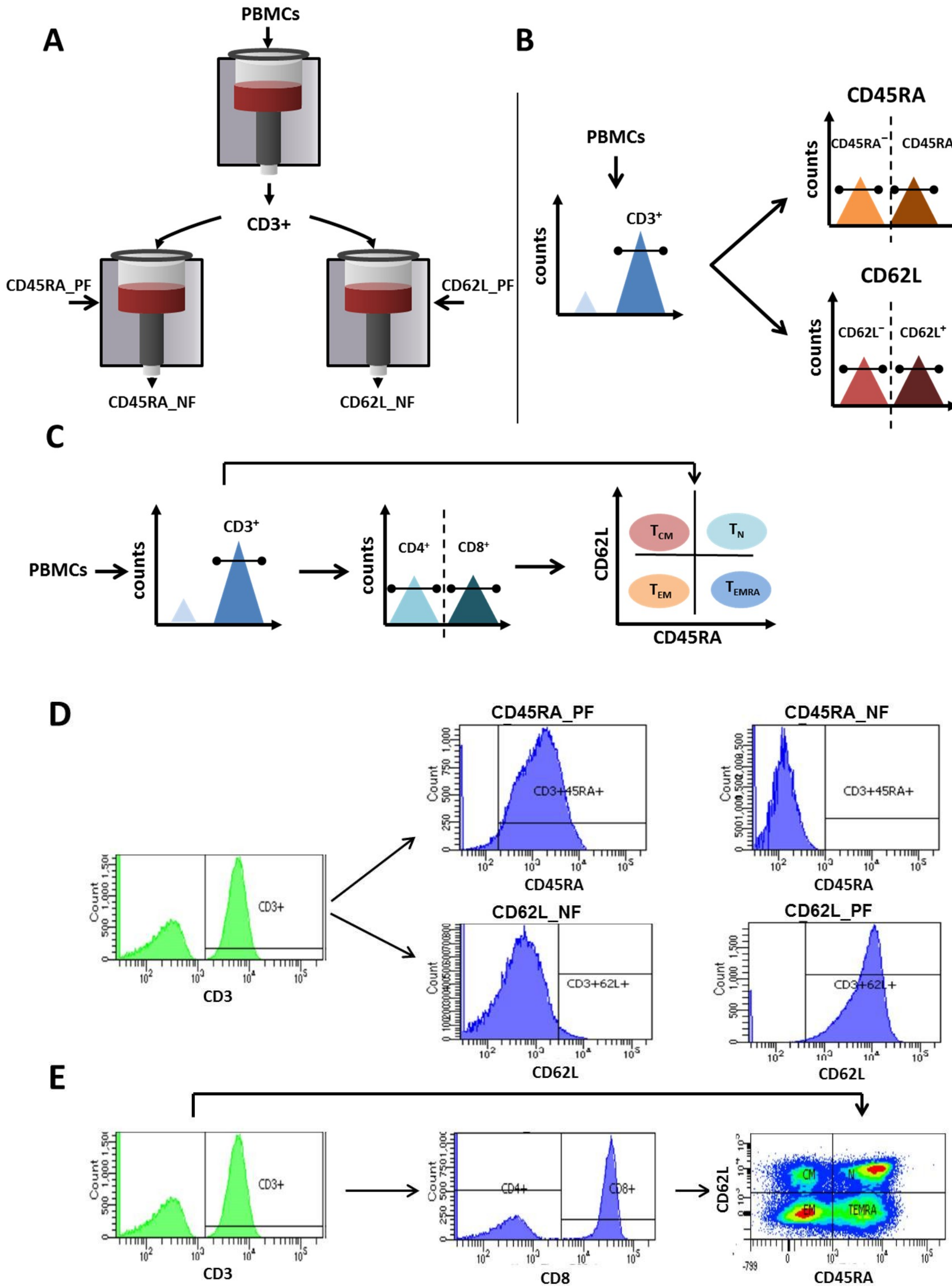

Supplementary Figure 1: Flow chart of T-cell isolation and phenotypic analysis. (**A**) Peripheral blood mononuclear cells (PBMCs) were isolated from CMV-seropositive donors by discontinuous-

gradient centrifugation. Untouched CD3+ T cells were enriched by magnetic cell sorting (MACS) using the Pan T-Cell Isolation Kit (Miltenyi Biotec). CD3+ T cells were collected as flow through. Bead-loaded non-T cells were collected from the columns as eluate and further used as a target cell population in the target cell-dependent ELISpot assay. PBMCs and CD3+ T cells were used as starting populations for naive T-cell depletion using CD45RA and CD62L immunomagnetic microbeads with LS+ and LD− columns, respectively. The positive fraction remained on the column while the negative fraction was collected as flow-through cells. Cell fractions obtained in this manner were divided into a memory fraction consisting of CD3+CD45RA− (CD45RA_NF) and CD3+CD62L− (CD62L_NF) T cells, and a naive fraction consisting of CD3+CD45RA+ (CD45RA_PF) and CD3+CD62L+ (CD62L_PF) T cells. (**B**) Gating strategy on frequency and phenotype of T-cell populations within T-cell fractions. This gating strategy was used to determine the frequencies of the different T-cell populations and fractions using different markers. At least 30,000 events were acquired in the live gate for each analysis. Gates were set based upon the scatter properties of lymphocytes, followed by the exclusion of doublets and gating on CD3+ T cells. The CD45RA_PF and CD45RA_NF fractions were not tested for the purity of CD62L, and the CD62L_PF and CD62L_NF were not tested for the purity of CD45RA. (**C**) After discriminating between CD4+ and CD8+ T cells, the frequencies and purities of CD45RA+/– as well as CD62L+/– cells of all the T-cell fractions were determined. Further gating was done on CD45RA vs. CD62L to determine the phenotype. T cells were characterized as follows: naive T cells (T_N; CD45RA+CD62L+), central memory T cells (T_{CM}; CD45RA−CD62L+), effector memory T cells (T_{EM}; CD45RA−CD62L−), and late effector memory T cells re-expressing CD45RA (T_{EMRA}; CD45RA+CD62L−). (**D**) Exemplary histogram plots for the gating strategy for the purity of CD45RA_PF and CD45RA_NF as well as that of CD62L_PF and CD62L_NF, respectively. (**E**) Exemplary histogram and dot plots for the gating strategy for frequency and phenotype of different T-cell populations.

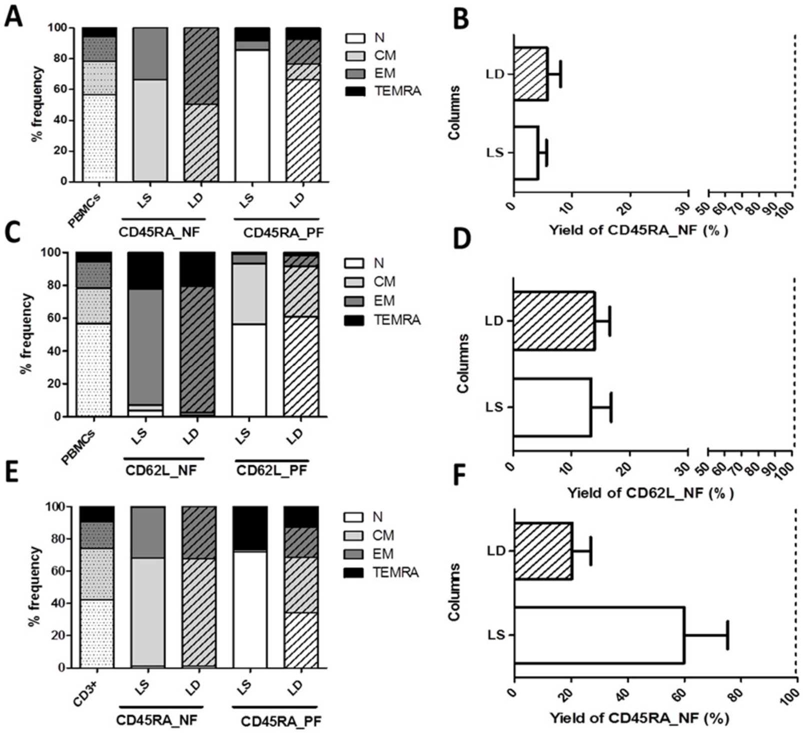

Supplementary Figure 2: Phenotypic composition of T-cell fractions and percentage yield of cells. (**A**, **C** and **E**) The phenotypic composition of memory and naive T-cell fractions obtained by naive T-cell depletion with LS+ and LD− columns using CD45RA and CD62L microbeads are shown. (**B**, **E** and

F) The recovery rate of memory T cells in the CD3+CD45RA_NF fractions and CD3+CD62L_NF fractions as percentage yield (the dotted line represents the expected yield) are shown. Depletion was performed on starting populations of PBMCs (**A–D**) and CD3+ T cells (**E–F**). The starting fractions are shown in columns with a dotted pattern. CD3+ T cells were isolated using the Pan T-Cell Isolation Kit (Miltenyi Biotec), and naive T-cell depletion was performed with LS+ (plain) or LD− (striated) columns. Percentage yields of T cells in the memory fractions were calculated as the number of CD45RA_NF /CD62L_NF cells times the % purity of CD45RA_NF/CD62L_NF T cells divided by the number of cells in the starting fraction multiplied by the % CD3+CD45RA−/CD62L− cells within starting fraction x 100. The expected yield was calculated to 100% purity of the CD45RA_NF fraction (**B** and **D**) and CD62L_NF (**F**) in the starting fraction.

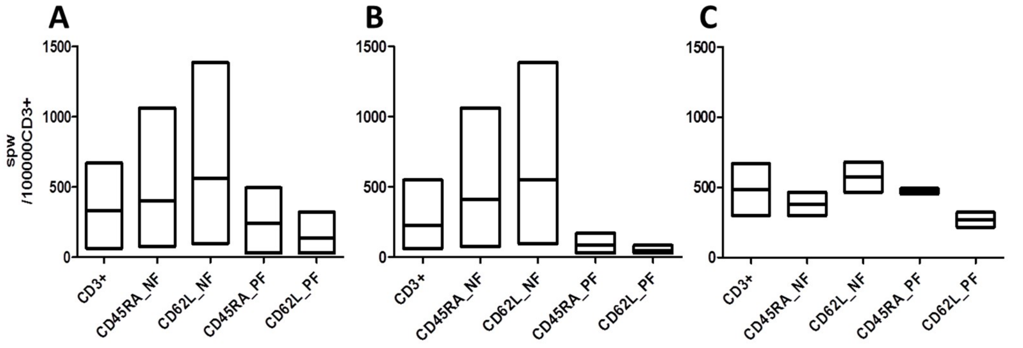

Supplementary Figure 3: T-cell response to ppCMV_pp65 as determined by ELISpot assay with an effector: target ratio of 2:1. T-cell responses against ppCMV_pp65 among the different T-cell fractions were evaluated with a target cell-independent ELISpot assay. Shown are the T-cell responses of (**A**) all donors (ALL, n=5), (**B**) donors with expected response (DER, n=3) and (**C**) donors with unexpected response (DUR, n=2). The lines within the boxes indicate mean values.

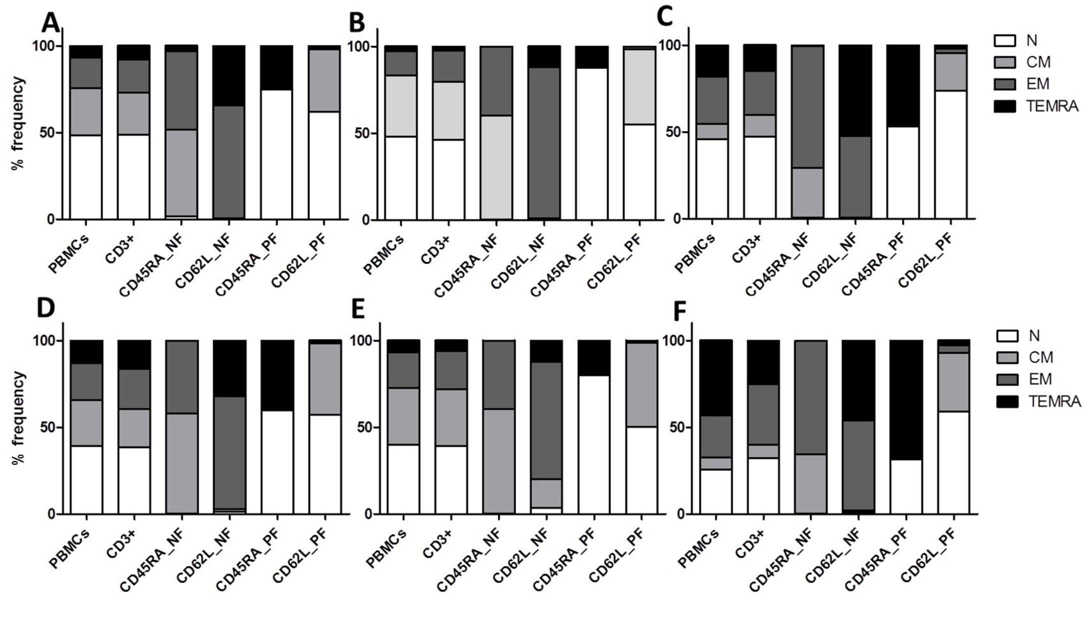

Supplementary Figure 4: Phenotype frequencies in the CD3+, CD4+ and CD8+ T-cell fractions. On top phenotype frequencies (%) of (**A**) CD3+ T cells, (**B**) CD4+ T cells and (**C**) CD8+ T cells in donors with expected response (DER) are shown. On the bottom phenotype frequencies of (**D**) CD3+ T cells, (**E**)

$CD4^+$ T cells, and (**F**) $CD8^+$ T cells in donors with unexpected response (DUR) are shown. Data represent the mean of six DER or DUR donors, respectively.

Supplementary tables

Supplementary Table 1A: Comparison of naive T-cell depletion strategies from PBMCs using LS^+ and LD^- columns. Shown are T-cell phenotype frequencies, T-cell counts, initial cell numbers and yields in the different T-cell fractions obtained from three donors using CD45RA and CD62L microbeads on a starting population of PBMCs. Phenotype frequencies (percentages) are represented as mean with range (minimum – maximum).

	Start (PBMC)	CD45RA+	CD45RA−	CD45RA_NF	Starting number of cells ($\times 10^6$)	Cells recovered in CD45RA−($\times 10^6$)	Yield (%)	CD45RA_PF
**LS+**								
**CD3+**	43.3(40.3-47.8)	26.93 (23.7-32.0)	73.1 (68.0-76.40)	28.13 (20.5-36.9)	14.62 (13.6-15.28)	2.03 (1.0-2.80)	4.2 (1.3-5.7)	64.5 (60.60-67.10)
T_N	56.8 (44.6-65.4)			0.37 (0.30-0.40)				85.33 (82.2-91.4)
T_{CM}	21.37 (15.6-26.5)			66.0 (57.60-70.80)				0.77 (0.30-1.30)
T_{EM}	16.3 (11.0-23.40)			33.6 (28.80-42.0)				5.47 (0.50-11.10)
T_{EMRA}	5.57 (3.1-8.1)			0.03 (0.0-0.10)				8.43 (6.10-12.40)
**LD−**								
**CD3+**	43.3(40.3-47.8)	26.93 (23.7-32.0)	73.1 (68.0-76.4)	23.87 (16.6-28.8)	14.62 (13.6-15.28)	3.2 (1.2-4.6)	5.8 (1.3-8.9)	61.9 (60.10-64.40)
T_N	56.8(44.6-65.4)			0.47 (0.40-0.50)				66.63 (52.3-79.4)
T_{CM}	21.37(15.6-26.5)			50.07 (44.2-60.20)				10.1 (0.30-16.10)
T_{EM}	16.3(11.0-23.4)			49.4 (39.20-55.20)				15.83 (10.6-22.0)
T_{EMRA}	5.57(3.1-8.1)			0.03 (0.0-0.10)				7.43 (3.0-9.70)

	Start (PBMC)	CD62L+	CD62L-	CD62L _NF	Starting number of cells ($\times 10^6$)	Cells recovered in CD62L− ($\times 10^6$)	Yield (%)	CD62L_PF
**LS+**								
**CD3+**	43.3 (40.3-47.8)	78.1 (71.1-82.4)	21.8 (17.6-28.9)	29.0 (22.80-35.60)	4.37 (3.5-5.78)	1.2 (0.0-2.4)	13.3 (9.5-20.3)	46.23 (38.7-55.2)
T_N	56.8 (44.6-65.4)			3.87 (2.70-5.10)				56.17 (45.6-74.1)
T_{CM}	21.37 (15.6-26.5)			3.27 (1.10-5.40)				37.03 (24.4-49.8)
T_{EM}	16.3 (11.0-23.4)			70.9 (50.50-85.70)				5.7(0.9-14.90)
T_{EMRA}	5.57 (3.1-8.1)			21.9 (5.10-45.70)				1.2(0.20-2.70)
**LD−**								
**CD3+**	43.3(40.3-47.80)	78.1 (71.1-82.4)	21.8 (17.6-28.9)	25.8(23.40-29.80)	4.37 (3.5-5.78)	2.2 (2.2-2.4)	13.9 (9.2-18.3)	50.2 (47.5-54.6)
T_N	56.8(44.6-65.4)			0.87(0.60-1.10)				60.9 (49.9-70.3)
T_{CM}	21.37(15.6-26.5)			1.7(0.5-2.6)				30.6 (21.6-41.1)
T_{EM}	16.3(11.0-23.4)			77(49.5-90.9)				6.73 (5.1-7.6)
T_{EMRA}	5.57(3.1-8.1)			20.5(6.0-49.2)				1.73 (0.9-3.0)

Supplementary Table 1B: Comparison of naive T-cell depletion strategies from CD3+ T cells using LS+ and LD− columns. Shown are T-cell phenotype frequencies, T-cell counts, initial cell numbers and yields in the different T-cell fractions obtained from three donors using CD45RA microbeads on a starting population of CD3+ T cells. Phenotype frequencies are expressed as mean percentages (%) with range (minimum – maximum).

	Start (CD3+)	CD45RA+	CD45RA−	CD45RA_NF	Starting number of cells (×10^6)	Cells recovered in CD45RA−(× 10^6)	Yield (%)	CD45RA_PF
**LS+**								
**CD3+**	97.1 (96.3-98.3)	41.2(35.7-45.8)	58.8 (54.1-64.4)	98.8 (98.6-99)	5.9 (5.4-6.4)	3.5 (2.0-5.0)	59.92 (32.2-85.4)	97.6(97.3-97.8)
T_N	42.2 (31.2-52.4)			0.9 (0.4-1.9)				71.8(55.5-81.7)
T_{CM}	31.9 (27.9-38.9)			67.1 (53.7-76.6)				1.47(1.4-1.6)
T_{EM}	16.5 (11-26.6)			31.37 (22.7-43.0)				0.93(0.7-1.4)
T_{EMRA}	9.3 (6.7-14.3)			0.57 (0.1-1.4)				25.8(16.0-41.8)
**LD−**								
**CD3+**	97.1 (96.3-98.3)	41.2(35.7-45.8)	58.8 (54.1-64.4)	96.67 (93.4-98.5)	8.75(5.78-10.82)	1.67(1.0-2.1)	20.34 (9.67-32.25)	47.1(35.8-54.7)
T_N	42.2 (31.2-52.4)			1.2 (0.7-1.8)				34.27(24.4-46.6)
T_{CM}	31.9 (27.9-38.9)			66.6 (47.7-78.0)				34.33(32.5-35.3)
T_{EM}	16.5 (11-26.6)			31.87 (21.2-50.2)				18.53(10.1-31.6)
T_{EMRA}	9.3 (6.7-14.3)			0.03 (0.0-0.10)				12.83(8.1-19.0)

Supplementary Table 2A: Phenotype frequencies, T-cell counts and cellular composition in donors with expected response (DER). Shown are T-cell phenotype frequencies and T-cell counts in the different T-cell fractions in donors with expected response. Phenotype frequencies within the $CD3^+$, $CD4^+$ and $CD8^+$ T-cell fractions are expressed as mean percentages with range (minimum – maximum). Data represent the mean of six independent determinations.

	**PBMC**	**CD3+**	**CD45RA_NF**	**CD62L_NF**	**CD45RA_PF**	**CD62L_PF**
**CD3+ [%]**	58.65(32.7-73.8)	96.73(93.0-97.9)	98.4(96.7-99.7)	96.12(95.0-98.0)	95.68(89.4-98.7)	96.95(91.5-98.8)
T_N [%]	48.57(19.6-63.2)	48.9(23-65.80)	1.6(0.00-8.30)	0.48(0.00-1.10)	74.92(61.80-91.90)	61.78(32.20-74.50)
T_{CM} [%]	26.9 (15.3-49.0)	23.93(13.9-44.80)	50.23(31.40-70.20)	0.12(0.00-0.40)	0.63(0.30-1.10)	36.12(25.30-67)
T_{EM} [%]	17.82(9.3-28.5)	19.15(10.90-28.80)	45.15(29.40-51.60)	65(34.50-89.30)	0.75(0.30-1.80)	1.22(0.20-3.10)
T_{EMRA} [%]	6.7(1.4-15.60)	8.05(3.10-13.00)	3.05(0.00-17.80)	34.35(10.60-64.50)	23.68(7.20-36.60)	0.88(0.00-2.70)
**CD4+ [%]**	78.2(59.9-93.2)	65.05(55.2-74.7)	79.97(74.8-84.0)	44.58(32.6-55.3)	58.67(46.2-71.6)	71.03(60.6-80.4)
T_N [%]	48.07(13.6-60.8)	46.22(13-63)	0.45(0.0-1.8)	0.52(0.0-1.0)	87.78(80.5-96.7)	87.78(80.5-96.7)
T_{CM} [%]	35.02(24.2-72.0)	33.42(22.5-59.7)	59.53(53.6-74.3)	0.48(0.0-1.5)	0.95(0.4-1.9)	43.12(27.1-82.4)
T_{EM} [%]	14.27(10.9-18.8)	17.95(10.0-27.1)	39.7(25.5-46)	87.05(79.3-98.8)	0.87(0.2-2.0)	1.37(0.2-4.0)
T_{EMRA} [%]	2.67(0.4-4.4)	2.4(0.2-4.1)	0.3(0.00-1.6)	11.97(1.2-20.3)	11.97(1.2-20.3)	10.4(2.5-18.8)
**CD8+ [%]**	18.67(6.8-35.0)	33.38(25.1-42.2)	19.42(16.0-24.3)	52.28(44.7-63.0)	39.47(28.0-49.9)	28.13(19.9-37.0)
T_N [%]	45.85(17.4-81.9)	47.17(22.2-80.8)	0.67(0.2-1.5)	0.65(0-1.6)	53.03(25.2-89.6)	73.68(34.4-93.7)
T_{CM} [%]	8.92(2.1-25.8)	12.68(1.8-35.7)	28.7(13.3-62.8)	0.067(0.0-0.2)	1.32(0.1-6.2)	21.73(2.8-59.9)
T_{EM} [%]	27.08(3.2-43.4)	25.12(2.9-48.6)	70.08(36.6-86.2)	46.88(7.1-77.7)	0.7(0.1-2.0)	2.57(0.3-4.9)
T_{EMRA} [%]	18.12(7.4-36.3)	15.07(3.1-33.6)	0.5(0.1-1.5)	52.37(22.2-91.2)	44.95(9.6-68.3)	1.97(0.1-5.4)
**CD4/8 ratio**	4.19	1.95	4.12	0.85	1.49	2.53
**Tregs [%]**	5.9 (5.6-6.2)	5.33 (4.8-6.0)	6.77 (3.1-9.4)	2.47 (1.1-4.2)	4.9 (1.7-6.9)	6.27 (6.0-6.5)
**γδ T cells [%]**	2.63 (0.5-4.6)	1.3 (0.5-2.2)	1.53 (0.3-2.9)	1.5 (0.6-3.1)	2.45 (0.5-3.5)	2.73 (0.5-5.7)

Supplementary Table 2B: Phenotype frequencies, T-cell counts and cellular composition in donors with unexpected response (DUR). Shown are T-cell phenotype frequencies and T-cell counts in the different T-cell fractions in donors with unexpected response. Phenotype frequencies within the $CD3^+$, $CD4^+$ and $CD8^+$ T-cell fractions are expressed as mean percentages with range (minimum – maximum). Data represent the mean of six independent determinations.

	PBMC	CD3+	CD45RA_NF	CD62L_NF	CD45RA_PF	CD62L_PF
**CD3+ [%]**	50.38(34.2-57.6)	95.57(92.4-98.4)	98.1(97.5-99)	96.03(92-97.4)	93.27(88.3-95.6)	94.63(90.4-97.6)
T_N [%]	39.13(26.2-55.5)	38.63(27.2-50.2)	0.17(0-0.5)	1.32(0.1-6.8)	59.65(35.0-76.5)	57.2(47-67.9)
T_{CM} [%]	26.48(15.7-42.6)	21.98 (10.6-32.2)	57.93(41.7-74.2)	1.52(0-8.6)	0.5(0.2-0.8)	41.32(30.2-51.9)
T_{EM} [%]	21.22(16.7-30)	22.95(13.2-31.7)	41.82(25.6-58.2)	65.15(49.7-78.9)	0.8(0.3-1.7)	0.87(0.3-67.9)
T_{EMRA} [%]	13.15(2.9-28.1)	16.43(7.6-32.7)	0.07(0.0-0.2)	31.98(11.1-50)	39.05(22.4-62.6)	0.63(0.3-1.4)
**CD4+ [%]**	76.22(51.3-90.4)	65.77(51.3-82.9)	81.18(74.8-90.7)	44.4(37.7-56.4)	55.08(36.8-76.1)	72.92(53.4-89.2)
T_N [%]	40.1(28.2-52.8)	39.17(28.4-49.1)	0.2(0-0.6)	3.62(0.1-11)	3.61667(0.1-11)	79.78(64.2-89.6)
T_{CM} [%]	32.5 (22.1-44.7)	32.62(16.6-44.4)	60.4(44.9-71.7)	16.35(0-77.8)	1.3(0.1-3.3)	48.32(40.7-55.7)
T_{EM} [%]	20.55(16.6-35.8)	22.25(16.2-35.3)	39.32(28.2-55)	67.53(11.5-98.2)	1.23(0.3-2.6)	1.18(0.4-3.6)
T_{EMRA} [%]	6.87(1.5-20.8)	6(1.6-21.2)	0.08(0.0-0.3)	12.48(0.6-45.2)	17.67(7.1-33.6)	0.23(0.1-0.8)
**CD8+ [%]**	21.5(6.3-48.7)	33.4(15.9-48.7)	18.2(9-25.2)	53.95(42-62.3)	43.15(22.1-63.2)	25.93(9.9-46.6)
T_N [%]	25.55(0.8-63.6)	32.1(22.1-52.8)	0.22(0.1-0.7)	1.18333(0.1-3.6)	31.42(17.4-43.5)	59.13(34.3-76.7)
T_{CM} [%]	7.07 (0.2-13.2)	7.83(5.4-12.5)	34.43(17.2-61.3)	0.8(0.0-4.0)	0.45(0.1-1.0)	0.45(0.1-1)
T_{EM} [%]	24.38(5.2-51.5)	34.8(21.5-48.89)	65.07(38.4-81.8)	51.78(14.7-72.1)	1.18(0.7-2.1)	4.37(1.1-13.3)
T_{EMRA} [%]	43.03(7.8-72.1)	25.25(9.3-51)	0.28(0.0-0.9)	46.22(20.3-84.9)	66.93(55.3-81.7)	2.7(0.7-6.4)
**CD4/8 ratio**	3.55	1.97	4.46	0.82	1.28	2.81
**Tregs [%]**	6.97 (3.3-9.3)	7.93 (5.7-11.5)	8.47 (1.2-13.4)	6.37 (2.7-12.1)	6.5 (4.5-9.7)	4.87 (2.5-7.4)
**γδ T cells [%]**	3.23 (1.8-4.1)	2.17 (1.4-3.2)	0.8 (0.6-1.0)	2.93 (1.8-4.3)	2.93 (1.9-3.8)	1.87 (1.5-2.3)
